# Comparing Small-Angle X‑ray Scattering Approaches for Quantifying Magnetic-Field-Induced Orientation of Superparamagnetic Iron Oxide Nanoparticles

**DOI:** 10.1021/acs.langmuir.6c04285

**Published:** 2026-10-01

**Authors:** Andreas F. Thünemann

**Affiliations:** 42220 Bundesanstalt für Materialforschung und -prüfung (BAM), Unter den Eichen 87, 12205 Berlin, Germany

## Abstract

The magnetic-field-induced orientation of superparamagnetic multicore iron oxide nanoparticles, exemplified by ferucarbotran (Resovist), was quantified by small-angle X-ray scattering (SAXS). Scattering patterns were recorded as a function of magnetic field strength and particle concentration and analyzed using the Maier–Saupe orientational distribution function, originally introduced for nematic liquid crystals. This analysis yielded the second-order orientational order parameter, which was compared with values obtained from a circular-function approach and a model-free evaluation. The close agreement between the different procedures demonstrates that SAXS provides a reliable basis for quantifying field-induced orientational order in dispersions of multicore iron oxide nanoparticles. Beyond its relevance for colloid and nanomaterials research, this method comparison may also be useful for biomedical applications of magnetic nanoparticles, for which reproducible characterization and standardized analysis protocols are essential. In the longer term, automated SAXS workflows, potentially supported by artificial-intelligence-assisted data evaluation, could further improve the speed, robustness, and comparability of orientation analyses in anisotropic nanostructures and colloidal systems.

## Graphical abstract

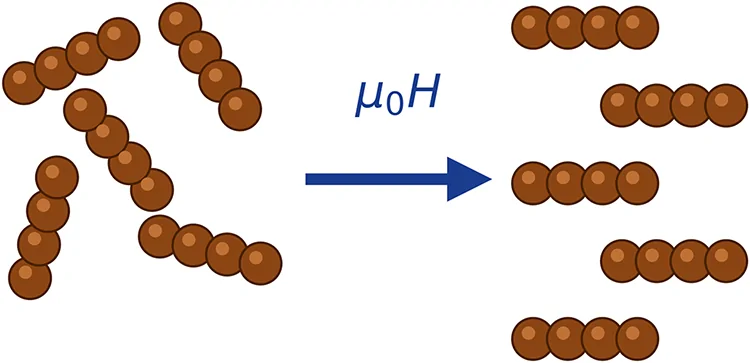

## Introduction

Superparamagnetic iron oxide nanoparticles have attracted broad interest because their magnetic response is highly sensitive to particle composition, crystal structure, size, and surface chemistry.^1^ These features are particularly important in biomedical applications, where colloidal stability, biocompatibility, and nanoparticle–medium interactions must be carefully controlled. Beyond biomedicine, magnetic nanoparticles are also widely used in environmental applications, exploiting magnetic separation for pollutant adsorption and degradation.^2^ Magnetic particle imaging (MPI), an emerging method for mapping the spatial distribution of superparamagnetic nanoparticles, holds strong promise for in vivo diagnostics.^3^ Examples include imaging of atherosclerotic pathobiology,^4^ in vivo tracking of macrophages,^5^ and theranostic approaches that integrate imaging with therapy.^6^ Related theranostic platforms based on magnetic graphitic nano- and microcapsules have also been explored for combined diagnostic and therapeutic functions.^7^ MPI performance is largely determined by the nanoparticles’ physicochemical and magnetic properties, including core size, size distribution, magnetic anisotropy, and relaxation dynamics.^8^ Beyond imaging, the field responsiveness of embedded magnetic nanoparticles is also exploited directly in biomedical engineering, for example in magneto-responsive hydrogel microcarriers for controlled cell seeding.^9^

Ferucarbotran, a commercially available formulation of superparamagnetic iron oxide nanoparticles marketed as Resovist in Japan, has been widely used in magnetic resonance imaging (MRI) and MPI and is also frequently employed as a reference material.^10,11^ Originally developed by Schering, ferucarbotran was the second clinically approved superparamagnetic iron oxide formulation introduced for contrast-enhanced liver MRI.^12,13^ The formulation has been used clinically for more than a decade. Ferucarbotran consists of stable multicore aggregates formed from primary particles with core radii ranging from 4 to 13 nm.^14,15^ Because the formulation contains a large amount of carboxydextran as a colloidal stabilizer, high-resolution electron microscopy is difficult, and the available TEM images therefore provide only limited structural detail.^16^ Löwa and co-workers investigated ferucarbotran by hydrodynamic and magnetic fractionation combined with detailed magnetic characterization by magnetorelaxometry.^15^ This approach provided access to the effective magnetic anisotropy of the particle fractions. The same group also examined the influence of hydrodynamic fractionation on MPI performance^17^ and later compared ferucarbotran with newly developed multicore nanoparticles.^10^ Mohn et al. presented a comparative characterization of four MPI tracers—Resovist, Resotran, Perimag, and Ferrotran.^18^ Given the extensive work devoted to understanding the structure and properties of these similar particle systems, it is notable that the orientational behavior of these nanoparticles in a static magnetic field has not yet been examined. The present study addresses this question using small-angle X-ray scattering (SAXS). To this end, SAXS measurements were performed on ferucarbotran (Resovist)^12^ exposed to static magnetic fields of varying strengths. For a wide overview of small-angle scattering to guide functional magnetic nanoparticle design, we refer to Honecker et al.^19^

## Experimental Section

### Materials

Ferucarbotran (Resovist, stored in two glass vials labeled E249/BA0171 25, 8044-98, 17.03.98, Schering, Berlin, Germany) was used as received.

### SAXS Measurements

SAXS measurements were performed using a customized Xeuss 2.0 instrument (Xenocs, France).^20^ The instrument was equipped with a microfocus copper X-ray source and multilayer optics for beam collimation and monochromatization, providing Cu Kα radiation with a wavelength of *λ*_Cu_ = 0.154 nm. A dedicated sample holder was designed to position a flow-through capillary in a well-defined magnetic field, as shown in Figure S1. The magnetic field was generated by two cylindrical N40 permanent magnets mounted on a rail system. The field strength at the capillary position was adjusted by sliding the magnets along the rail. Figure S2 shows the magnetic field strength, expressed as μ_0_ *H*, as a function of the distance, *d*, between the magnets and the center of the capillary. The values of μ_0_ *H* used for the SAXS measurements, together with the corresponding distances *d*, are listed in Table S1. Within the X-ray-illuminated volume of the capillary (inner diameter 1 mm, matching the 1 mm circular footprint of the primary beam), the magnetic field varies by less than 3% of the local value at field settings ≤ 30 mT and by up to 4.3% at the highest field setting (118 mT), where the local gradient d*B*/d*d* is largest (5.0 mT/mm at the 50 mm working distance). These values were estimated from the field-versus-distance calibration underlying Figure S2 and Table S1. The magnetic field strengths investigated here were restricted to a range in which SAXS anisotropy is observed without macroscopic phase separation. Upon removal of the magnetic field, the SAXS pattern returned to its original isotropic form, confirming reversibility, as expected from Brownian motion of these small, superparamagnetic particles.

## Results and Discussion

SAXS measurements were performed on Resovist samples in the native formulation^6^ at an iron concentration of *c*_0_ = 0.5 mmol Fe mL^–1^ and on samples diluted with water to a concentration of *c*_1_ = 0.1*c*_0_. The two concentrations were selected to evaluate whether dilution affects the scattering behavior. The applied magnetic field strengths were 0, 4, 6, 8, 12, 18, 30, 53, 118, and 296 mT. For data analysis, the corresponding non-rounded field values were used. These field strengths correspond to mass magnetizations ranging from *M* = 0 to 82 A m^2^ kg^–1^ relative to the iron mass and span a broad portion of the magnetization curve shown in Figure S3. The magnetization data were taken from Yoshida et al.^11^ Comparable values have also been reported by Kratz et al.^10^

The X-ray transmission of the samples remained constant during SAXS measurements up to 118 mT, whereas an increase in transmission was observed at 296 mT and higher fields. This increase is consistent with magnetic separation of the nanoparticles from the irradiated region of the capillary. As the nanoparticles are displaced from the capillary center by the applied magnetic field, the particle concentration within the X-ray beam decreases, resulting in reduced absorption and, hence, increased transmission. For this reason, only SAXS patterns recorded at magnetic field strengths up to 118 mT were included in the subsequent analysis. A similar experimental strategy was employed by Huang et al. in a synchrotron SAXS study of the reversible rearrangement of magnetic nanoparticles.^21^

Visual inspection of the SAXS intensity contour maps shows an isotropic scattering pattern in the absence of an applied magnetic field, as expected and as shown in the left-hand panel of Figure 1. The radially averaged intensity is displayed in the right-hand panel of Figure 1 as blue circles and compared with data obtained on a line-focus instrument in an earlier study,^14^ shown as a black solid line. The close agreement between the two data sets confirms that the present measurements reproduce the previously reported SAXS signature of ferucarbotran. Ferucarbotran is composed of carboxydextran-stabilized superparamagnetic iron oxide multicore particles, in which several iron oxide nanocrystals form magnetically responsive clusters and chain-like aggregates.^18^ The primary iron oxide nanocrystals are approximately spherical and have radii of about 5 nm from SAXS^14^ and about 4 nm from TEM.^18^ TEM further revealed a non-uniform morphology of the multicore particles.^18^

**Figure 1:**
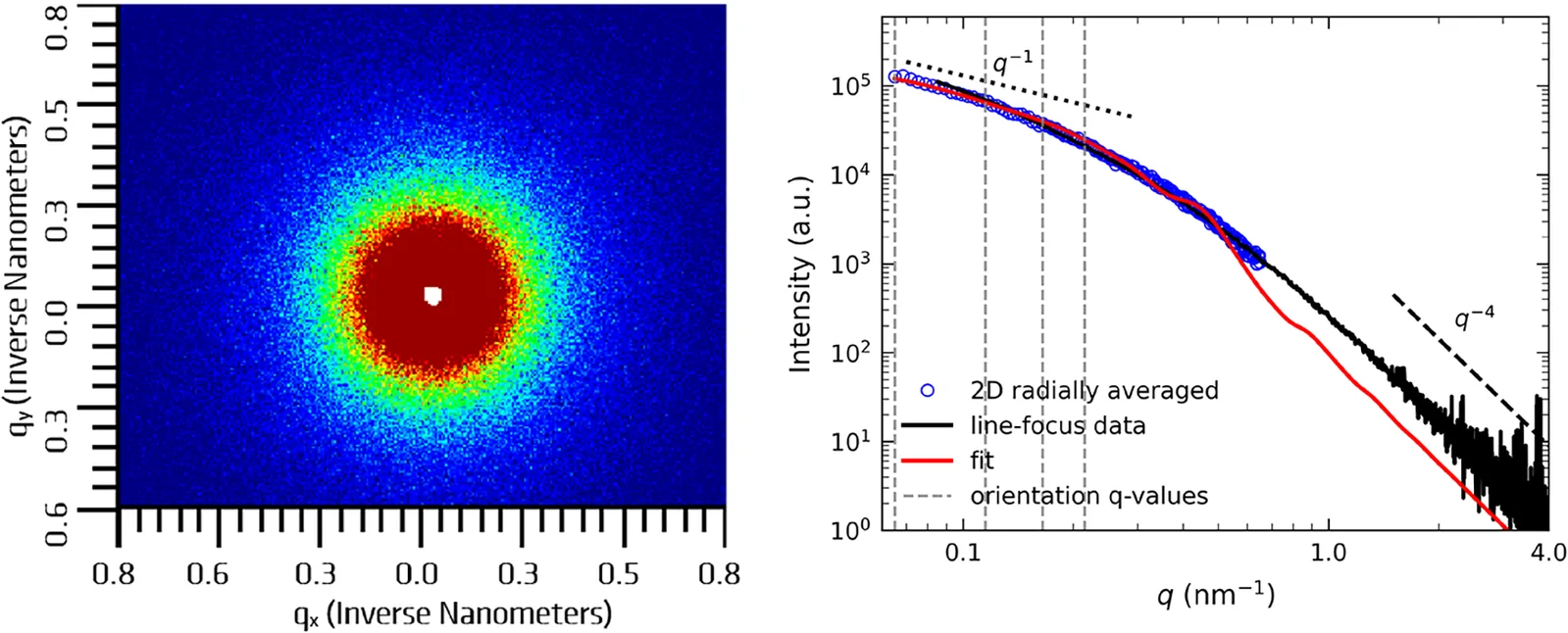
Left: SAXS intensity contour map recorded without an applied magnetic field for nanoparticles at their original concentration of *c*_0_ = 0.5 mmol Fe mL^–1^. Right: Corresponding radially averaged scattering intensity *I*(*q*) as a function of the scattering vector *q*. Blue open circles show the present 2D SAXS data, the black solid line shows line-focus SAXS data from an earlier study,^14^ and the red solid line represents the model fit. The dotted and dashed guide lines indicate approximate *q* ^–1^ scaling at low *q* and Porod scaling, *I*(*q*) ∝ *q* ^–4^, at high *q*, respectively. Vertical grey dashed lines mark the four *q*-values (0.065, 0.115, 0.165, and 0.215 nm^–1^) at which the azimuthal intensity profiles used for the orientational order analysis were extracted.

Despite this well-established structural heterogeneity, we sought to fit the isotropic SAXS curve using a simplified multicore nanoparticle model. As one possible description, we tested a multicore nanoparticle aggregate represented by a mass-fractal structure, which has previously been useful for interpreting SAXS data of similar systems.^22^ However, this mass-fractal multicore model did not adequately reproduce the present data. In particular, the low-*q* intensity scales approximately as *I*(*q*) ∝ *q* ^–1^, as indicated in the right-hand panel of Figure 1. At high *q*, the intensity approaches Porod behavior, *I*(*q*) ∝ *q* ^–4^.

Taken together, the scaling behavior at small and large *q* suggests a model that appears cylindrical at large length scales, while still consisting of smaller particles with a sharp interface with the surrounding medium. The simplest model that fulfills these requirements is a one-dimensional arrangement of nanoparticles, described by a 1D hard-sphere structure factor derived from the Percus–Yevick solution.^23^ This model is implemented in the SASfit software package, which was used for the analysis.^24^ In this description, the primary particles are treated as spheres with a Gaussian size distribution, a mean diameter of 10 nm, and a distribution width of 1.9 nm. Four such spherical particles are arranged linearly as a 1D paracrystal with a mean center-to-center distance of 15 nm and a standard deviation of 1 nm.

As shown in Figure 1, the best fit describes the present SAXS data well over the *q* range from 0.065 to 0.8 nm^–1^, corresponding to real-space distances of roughly 40 to 4 nm. The red line and the blue circles illustrate this agreement. At higher *q* values, corresponding to shorter distances, the calculated curve retains a similar overall shape but systematically underestimates the experimental intensity, as seen from comparison with the black curve. This deviation is most likely a consequence of the simplifying assumptions of the model and is not expected to affect the subsequent orientation analysis. For a more direct visualization of structural features, a Kratky representation (*q* ^2^·*I*(*q*) vs *q*) of the isotropic scattering data is provided in Figure S5.

One possible reason for the underestimated high-*q* intensity is that the surface-to-volume ratio, *S*/*V*, of the individual nanocrystals is larger than that of ideal spheres, possibly by about a factor of two. For a higher volume-weighted surface area, the Porod contribution, $I\left(q\right)\propto \frac{S}{V}q^{-4}$ , would be shifted upward on a double-logarithmic scale. Thus, deviations from spherical particle shapes or more complex surface morphologies could account for the remaining discrepancy at high *q*. Nevertheless, we retain this highly simplified model to avoid introducing additional fit parameters and the associated risk of overfitting. This choice is also supported by electron microscopy, which did not reveal evidence of a well-defined surface structure in the primary nanoparticles.^18^

The SAXS pattern becomes anisotropic when the sample is exposed to a magnetic field, as shown in Figure 2 at 118 mT. In this representation, the magnetic field direction is horizontal. With increasing magnetic field strength, the anisotropy of the SAXS pattern becomes more pronounced, as shown in Figure S4. Such anisotropic scattering patterns are characteristic of nanoscale structures with preferential orientation relative to the applied field. For objects with an approximately cylindrical scattering anisotropy, the intensity distribution indicates that their long axes are preferentially aligned with the magnetic field. As a first approximation, the nanoparticles are therefore described as field-induced linear arrangements oriented along the applied magnetic field, as illustrated schematically in the right-hand panel of Figure 2. Although this representation is necessarily simplified, it provides a useful starting point for extracting quantitative information on the orientational order of the corresponding nanoscale structures. In the following sections, the orientation of the multicore nanoparticles in the magnetic field is quantified from the anisotropic SAXS data. Reorientation of pre-existing multicore aggregates by the applied field, rather than the formation of new chains or elongation, is indicated by the lack of a systematic shift in low-*q* scattering with increasing field strength and the reversibility of the anisotropic pattern upon field removal. We emphasize that the number of four primary particles is not an independently determined structural parameter but rather the minimum aggregation number within the one-dimensional structure model required to reproduce the extent of the *q* ^–1^ scaling regime observed in the accessible *q*-range. It should therefore be interpreted as an effective, average description of a structurally heterogeneous particle population rather than a literal particle count. The orientation analysis in the following sections is restricted to *q* = 0.065–0.215 nm^–1^, a range in which the structure model reproduces the experimental isotropic scattering closely (Figure 1). The high-*q* deviation between model and data therefore does not affect the extracted orientation parameters.

**Figure 2:**
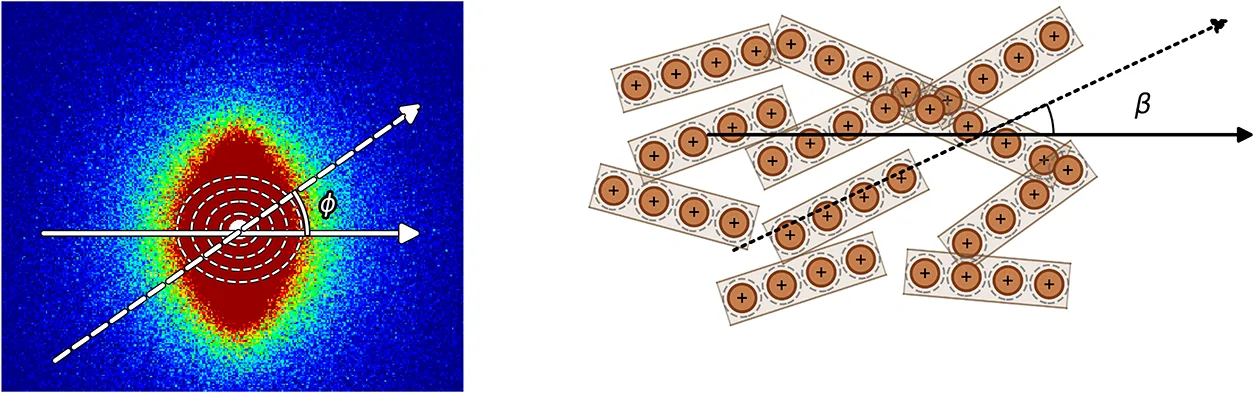
Left: SAXS intensity contour map of ferucarbotran particles recorded at a magnetic field strength of 118 mT. The magnetic field direction is horizontal and is indicated by the solid arrow. Dashed circles mark the four *q*-values (0.065, 0.115, 0.165, and 0.215 nm^–1^) at which the azimuthal intensity profiles used for the orientational order analysis were extracted. The dashed arrow denotes an arbitrary direction in the detector plane, and ϕ is the corresponding azimuthal angle with respect to the magnetic field direction. Right: Schematic representation of the simplified structural model used to describe the field-induced orientational order of the nanoparticles. The solid arrow denotes the magnetic field direction (director), whereas the dashed arrow indicates the main axis of a cylindrical particle arrangement. The angle β is defined as the angle between this axis and the magnetic field direction.

## Axial Orientation Distribution

Axial orientation describes the statistical distribution of the symmetry axes of anisotropic particles with respect to a reference direction, or director. In the case of nanoscale objects with cylindrical symmetry, the corresponding axial orientation distribution function, *f*(β), specifies the probability of finding the cylinder axis at an angle β relative to an aligning field, such as an external magnetic, electric, or mechanical field; determining this function from scattering data is therefore a central problem whenever such particles exhibit partial rather than perfect alignment. Representative examples include the orientational ordering of cellulose nanocrystals,^25^ the field-induced alignment of hematite spindles embedded in poly­(*N*-isopropylacrylamide) hydrogels,^26^ and the magnetic-field-driven orientational ordering of magnetic rod-like colloids.^27^

SAXS is well suited for determining *f*(β), although this orientation distribution is not measured directly. Rather, the primary experimental observable is the anisotropic scattering intensity *I*(*q*,ϕ), expressed as a function of the scattering-vector magnitude *q* and the azimuthal detector angle ϕ. Consequently, the relationship between the axial orientation distribution of the particles and the measured angular intensity profiles, in particular along the equatorial direction, must be evaluated within an appropriate scattering model.

Interestingly, two distinct and partly conflicting approaches have been reported in the literature: the method introduced by Kratky^28^ and the formulation developed by Leadbetter and Norris.^29^ The Kratky method has been used extensively in polymer scattering to describe orientation effects. In contrast, the Leadbetter–Norris approach is well established in the liquid-crystal literature for quantifying preferential orientational order.

Burger and Ruland demonstrated that the Kratky method provides the correct transformation. In contrast, the integral transformation proposed by Leadbetter and Norris is erroneous because it conflates the polar angle of the orientable unit with the polar angle describing the oriented ensemble.^30^ A discussion was provided by Agra-Kooijman et al.,^31^ who showed that the two approaches yield reasonably similar results for systems with sufficiently high orientational order. For weakly ordered systems, however, the Kratky method is required, as deviations between the two treatments become significant. The equation derived by Leadbetter and Norris^29^ is

1 $$I_{\mathrm{LN}}\left(\phi \right)=\int_{\beta =\phi }^{\pi /2}\frac{f\left(\beta \right)\sin\beta }{\cos^{2}\phi \sqrt{\tan^{2}\beta -\tan^{2}\phi }}d\beta$$

while Kratky^28^ derived

2 $$I_{K}\left(\phi \right)=\int_{\beta =\phi }^{\pi /2}\frac{f\left(\beta \right)\sin\beta }{\sqrt{\cos^{2}\phi -\cos^{2}\beta }}d\beta$$

Various functional forms have been employed to describe *f*(β), including expansions in Legendre polynomials,^32^ series of circular functions, and the Maier–Saupe distribution.^33^ In the following, we compare these three representations and apply them to the analysis of the SAXS patterns obtained from ferucarbotran samples exposed to different magnetic field strengths.

### Reconstruction of *f*(β) Using the Maier–Saupe Approach

For nematic liquid crystals, the Maier–Saupe distribution has proven useful for describing and quantifying orientational distributions. It has also been applied, for example, to the analysis of nematic order in dispersions of rod-like magnetic nanoparticles.^33^ The distribution is given by

3 $$f_{\mathrm{MS}}\left(\beta \right)=\frac{1}{Z}\exp\left(m\cos^{2}\beta \right)$$

where *Z* is a normalization constant and *m* is the Maier–Saupe distribution parameter.

Davidson et al. derived the scattering intensity corresponding to a Maier–Saupe orientational distribution. For classical mesophases composed of simple rod-like molecules, the resulting azimuthal intensity profile is given by^33^

4 $$I_{\mathrm{MS}}\left(\phi \right)=\frac{\exp\left(m\cos^{2}\phi \right)}{\sqrt{m}Z\cos\phi }\frac{\sqrt{\pi }}{2}\mathrm{erf}\left(\sqrt{m}\cos\phi \right)$$

The Maier–Saupe parameter, *m*, and its associated uncertainty, *u*_*m*_, were obtained by fitting azimuthal intensity profiles at fixed values of the scattering vector using eq 4. Reasonable-quality fits were obtained for both concentrations *c*_0_ and *c*_1_ over the full range of applied magnetic field strengths. Representative fits for *c*_0_ and *c*_1_, measured at applied field strengths of 4 mT and 118 mT, i.e., the lowest and highest fields of this study, are shown in Figure 3. An overview of the data and curves for the concentrations *c*_0_ and *c*_1_ at all fields and scattering vectors is provided in Figures S6–S9. A common feature is that the azimuthal peaks become narrower at higher field strength, corresponding to larger values of *m* and, hence, stronger orientational ordering, as expected. Furthermore, at a given field strength, the peaks are narrower for concentration *c*_0_ than for *c*_1_. This indicates a higher degree of alignment for the more concentrated sample. The resulting values of *m* were subsequently used to calculate the orientation distributions *f*(β) according to eq 3, as shown in Figure 4. For both *c*_0_ and *c*_1_, the distributions narrow systematically with increasing magnetic field strength.

**Figure 3:**
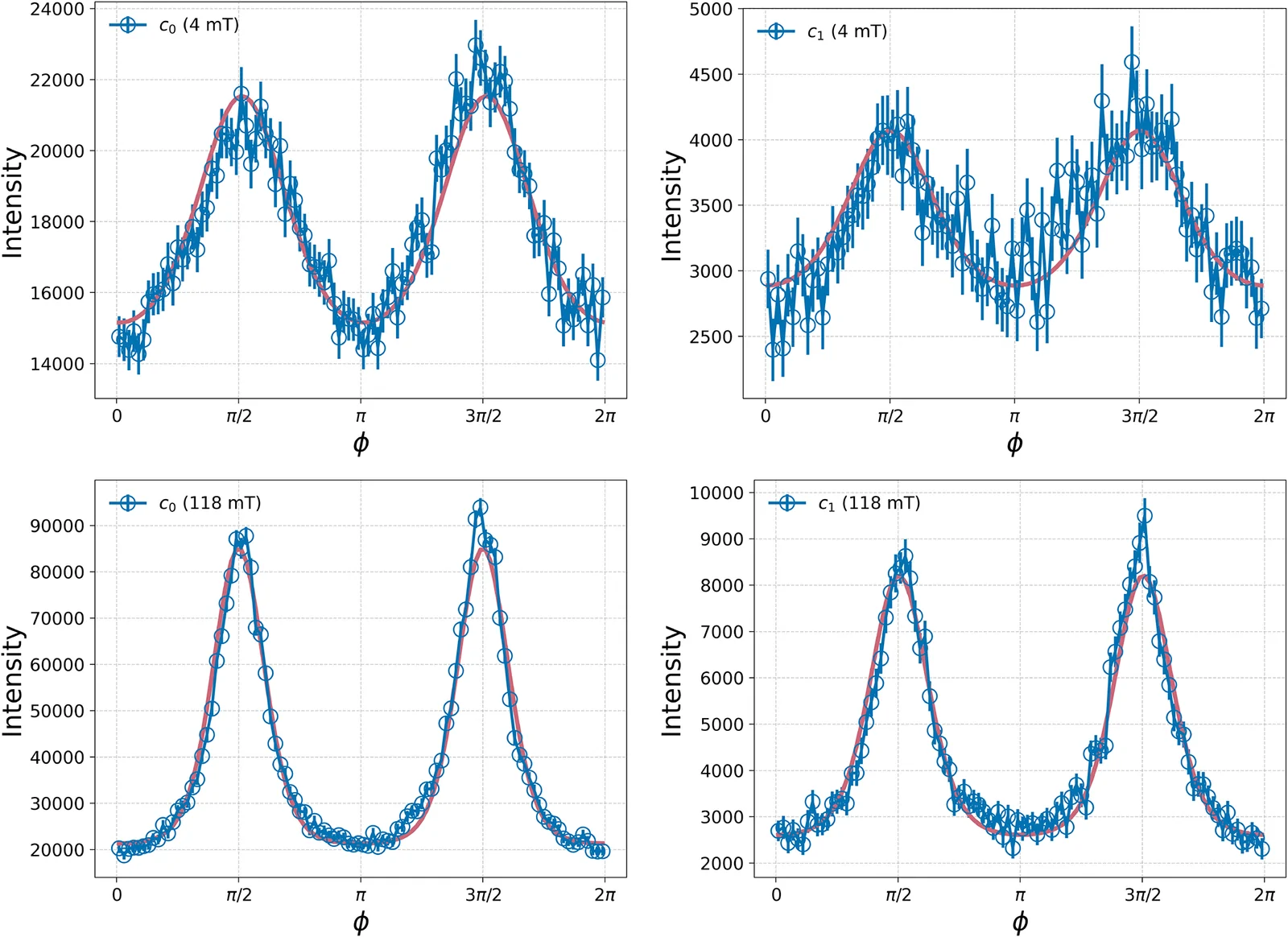
Azimuthal intensity distributions *I*(ϕ) (symbols) and the corresponding Maier−Saupe fits according to eq 4 (solid lines) for ferucarbotran at concentrations *c*_0_ and *c*_1_ (left- and right-hand panels, respectively). The scattering-vector magnitude was *q* = 0.215 nm^–1^.

**Figure 4:**
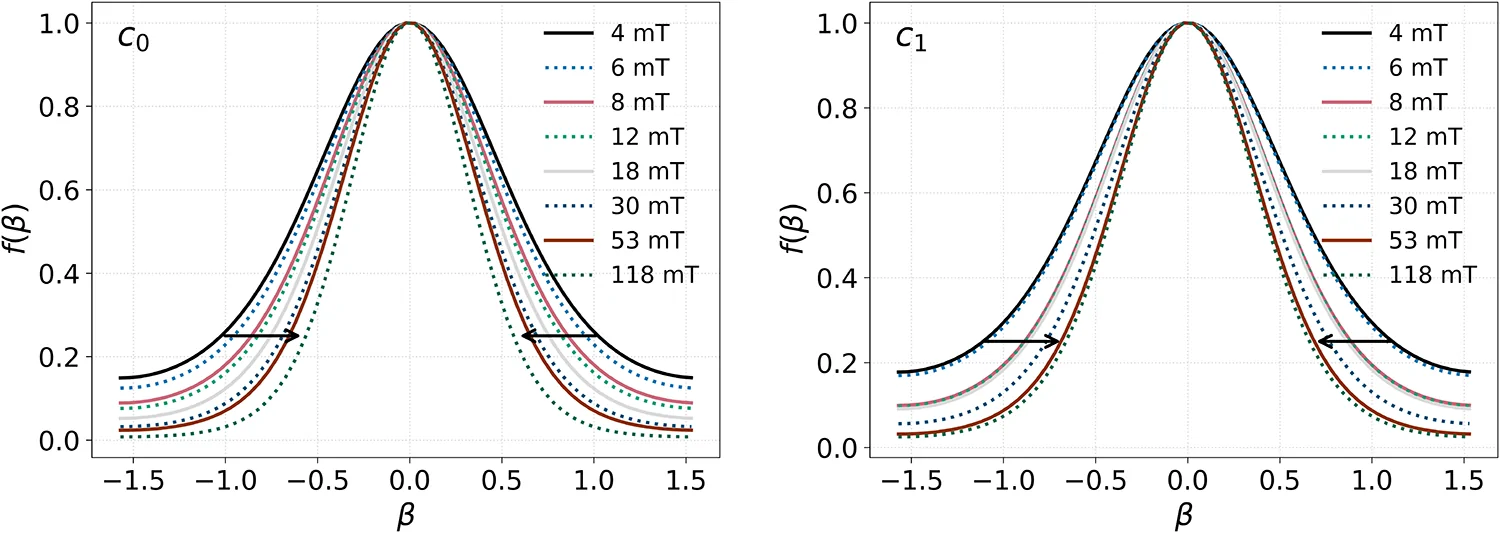
Orientation distribution functions *f*(β) obtained using the Maier−Saupe approach for particle concentrations *c*_0_ and *c*_1_ (left and right-hand figure, respectively). The *f*(β) curves are provided for magnetic field strengths ranging from 4 mT to 118 mT. The maxima are normalized to 1.

#### Order Parameter *S*

The Maier−Saupe parameter *m* is directly related to the order parameter *S*, which is also known as the Hermans order parameter.^34^ This order parameter was originally used for the evaluation of orientation in cellulose fibers from an X-ray fiber diagram and is defined as

5 $$S=\frac{1}{2}\left(3\left\langle \cos^{2}\beta \right\rangle -1\right)$$

Using *x* = cos­(β) leads to

6 $$\left\langle \cos^{2}\beta \right\rangle =\frac{\int_{0}^{1}x^{2}e^{mx^{2}}dx}{\int_{0}^{1}e^{mx^{2}}dx}$$

After integration, one obtains

7 $$S=\frac{3e^{m}}{2\sqrt{\pi m}\mathrm{erfi}\left[\sqrt{m}\right]}-\frac{1}{2}-\frac{3}{4m}$$

where erfi is the imaginary error function. Insertion of *m* in eq 7 provides values for the order parameter *S*. It should be noted here that this analysis applies to any nematic suspension of cylindrical moieties, provided that inter-particle positional correlations are negligible and that the Maier–Saupe distribution function is obeyed. Furthermore, different *f*(β) distributions can have the same value of *S*.

The uncertainty was propagated according to the law of propagation of uncertainty as described in Section 5.1.2 (eq 10) of the GUM^35^ as

8 $$u_{S}=\frac{3\pi \mathrm{erfi}{\left(\sqrt{m}\right)}^{2}+3\sqrt{\pi }e^{m}\sqrt{m}\left(2m-1\right)\mathrm{erfi}\left(\sqrt{m}\right)-6e^{2m}m}{4\pi m^{2}\mathrm{erfi}{\left(\sqrt{m}\right)}^{2}}u_{m}$$

The *S* values as a function of *m* were determined from the application of eqs 7 and 8 for the different magnetic field strengths. The results are shown in Figure 5 together with the theoretical *S*(*m*) curve. Therein, each *S*-value is the average of four values determined at different *q* values. We verified that *S* is independent of *q*, as expected (see Figure S10). An overview of the *m* and *S*-values is provided in Table S2.

**Figure 5:**
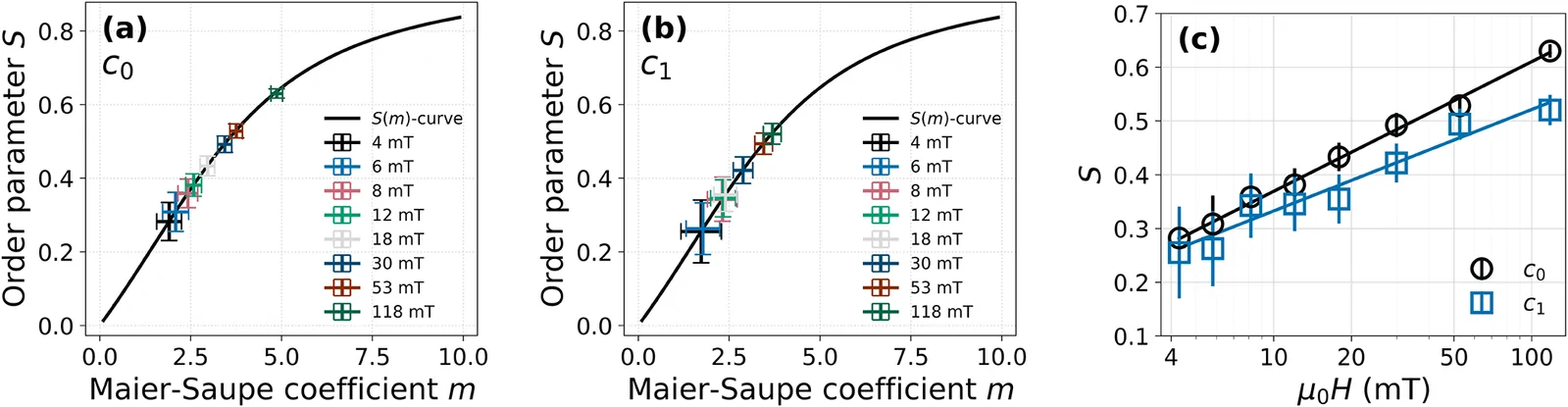
Order parameter *S* for ferucarbotran samples at concentrations *c*_0_ and *c*_1_. Panels (a, b) show *S* as a function of the Maier–Saupe coefficient *m* for *c*_0_ and *c*_1_, respectively. Theoretical *S*(*m*) curves are shown as solid lines, and measured values obtained at different applied magnetic fields are shown as symbols. Panel (c) shows *S* as a function of the magnetic field μ_0_ *H* for both concentrations, with *c*_0_ represented by black circles and *c*_1_ by blue squares. Linear fits to the data are shown as solid lines and yield slopes of 0.106 ± 0.003 for *c*_0_ and 0.081 ± 0.008 for *c*_1_.

When plotting the *S* values as a function of the magnetic field on a semi-logarithmic scale, the data points lie on a straight line. Hence, for quantification, the data were fitted by

9 $$S=a_{0}+a_{1}\ln\left(\mu_{0}H\right)$$

where μ_0_ *H* is given in units of mT. Data and curve fits obtained using eq 9 are shown in Figure 5(c). The best-fit intercepts are *a*_0_ = 0.127 ± 0.011 for *c*_0_ and *a*_0_ = 0.148 ± 0.029 for *c*_1_. The corresponding slopes are *a*_1_ = 0.106 ± 0.003 for *c*_0_ and *a*_1_ = 0.081 ± 0.008 for *c*_1_. The significantly larger value of *a*_1_ for *c*_0_ compared with *c*_1_ indicates that the order parameter increases more strongly with increasing field strength for *c*_0_. Equation 9 can therefore be used to estimate the hypothetical field strengths required to reach *S* = 1. For this theoretical limit of perfect alignment, a substantially lower field strength of 3.8 T is predicted for *c*_0_, whereas 37 T is required for *c*_1_.

### Reconstruction of *f*(*β*) Using a Circular-Functions Expansion

A series of circular functions of the form

10 $$f_{\mathrm{series}}\left(\beta \right)=\sum_{n=0}^{\infty }a_{2n}\cos^{2n}\beta$$

can be used for the present purpose without loss of generality, as derived by Davidson et al.^33^ Using this approach, Davidson et al. obtained an expression for the scattering intensity based on a well-known, but incorrect, integral equation; see eq 1. Later, Agra-Kooijman et al.^31^ employed the correct integral equation; see eq 2. This yields

11 $$I\left(\phi \right)=\sum_{n=0}^{\infty }a_{2n}\frac{\sqrt{\pi }\Gamma \left(1/2+n\right)}{2\Gamma \left(1+n\right)}\cos^{2n}\phi$$

The measured data must be normalized by a factor *N* such that

12 $$\frac{1}{N}\int_{0}^{\pi /2}I\left(\phi \right)\cos\phi d\phi =1$$

The coefficients in eq 11 therefore directly correspond to the coefficients in the expansion of *f*(*β*) given in eq 10. Experimentally, we evaluated the applicability of the finite series

13 $$I\left(\phi \right)=\frac{\pi }{2}\left[a_{0}+\frac{1}{2}a_{2}\cos^{2}\phi +\frac{3}{8}a_{4}\cos^{4}\phi +\frac{5}{16}a_{6}\cos^{6}\phi +\frac{35}{128}a_{8}\cos^{8}\phi \right]$$

and found that unambiguous fit results could only be obtained after applying suitable constraints. These constraints were imposed by setting *a*_4_ = 0 and *a*_6_ = 0, which reduces eq 13 to

14 $$I\left(\phi \right)=\frac{\pi }{2}\left[a_{0}+\frac{1}{2}a_{2}\cos^{2}\phi +\frac{35}{128}a_{8}\cos^{8}\phi \right]$$

The coefficients *a*_0_, *a*_2_, and *a*_8_ therefore remain as the only adjustable parameters. All curve fits are provided in Figures S11–S14. The fit parameters *a*_0_, *a*_2_, and *a*_8_ are shown in Figure S15, and the mean values are displayed in Figure 6. It can be seen that *a*_0_ decreases slightly as the field increases, *a*_2_ reaches a maximum around 20 mT, and *a*_8_ increases strongly. The shape of the curves is very similar for both concentrations, *c*_0_ and *c*_1_. Notably, the increase in *a*_8_ is substantially more pronounced at *c*_0_ than at *c*_1_. This suggests that nonlinear contributions to the magnetic-field-induced orientation are much more pronounced at the higher concentration. After applying eq 14, the orientation distribution function simplifies to

15 $$f_{\mathrm{series}}\left(\beta \right)=a_{0}+a_{2}\cos^{2}\beta +a_{8}\cos^{8}\beta$$

The resulting functions *f*_series_(β) are shown as a function of the magnetic field in Figure 7 for the concentrations *c*_0_ and *c*_1_. The narrowing of *f*(β) with increasing magnetic field strength is clearly visible, with a more pronounced effect observed for *c*_0_ than for *c*_1_. This behavior further indicates that nonlinear and collective particle effects contribute to the field-induced orientation.

**Figure 6:**
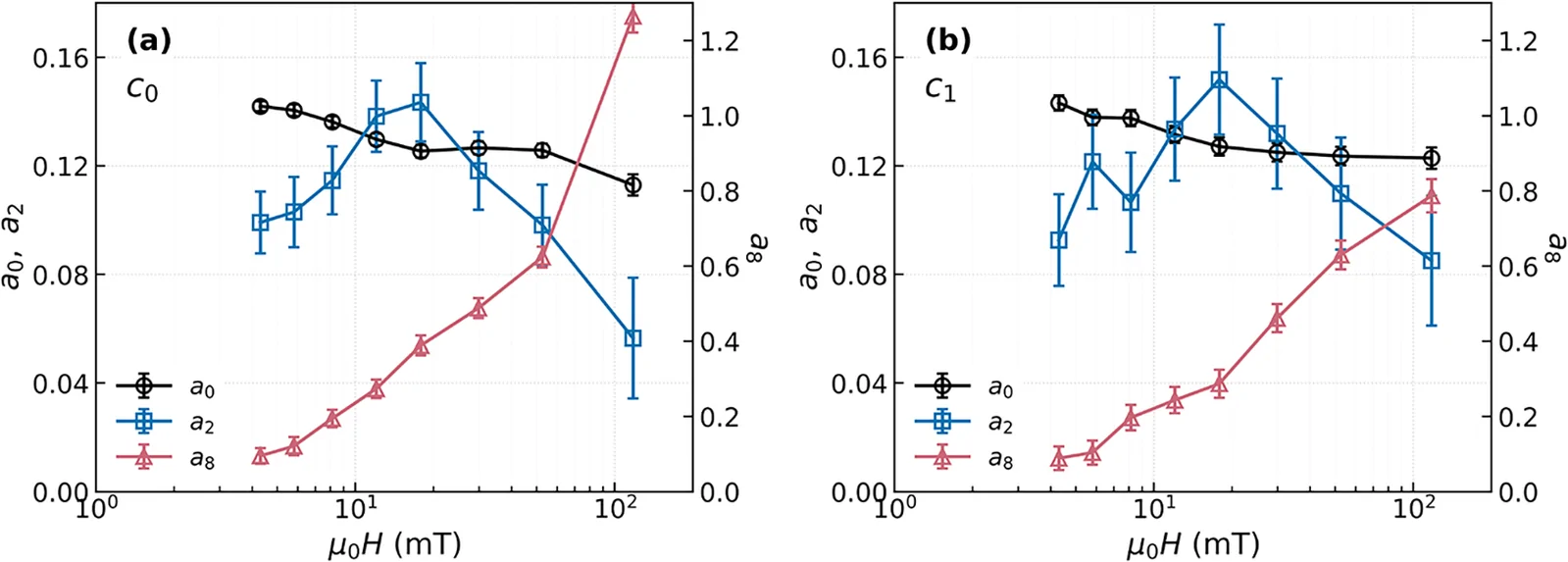
Circular-function approach. Magnetic-field dependence of the fitted expansion coefficients *a*_0_, *a*_2_, and *a*_8_ for particle concentrations *c*_0_ and *c*_1_. Panels (a, b) correspond to *c*_0_ and *c*_1_, respectively. The coefficients *a*_0_ and *a*_2_ are plotted on the left ordinate, whereas *a*_8_ is plotted on the right ordinate.

**Figure 7:**
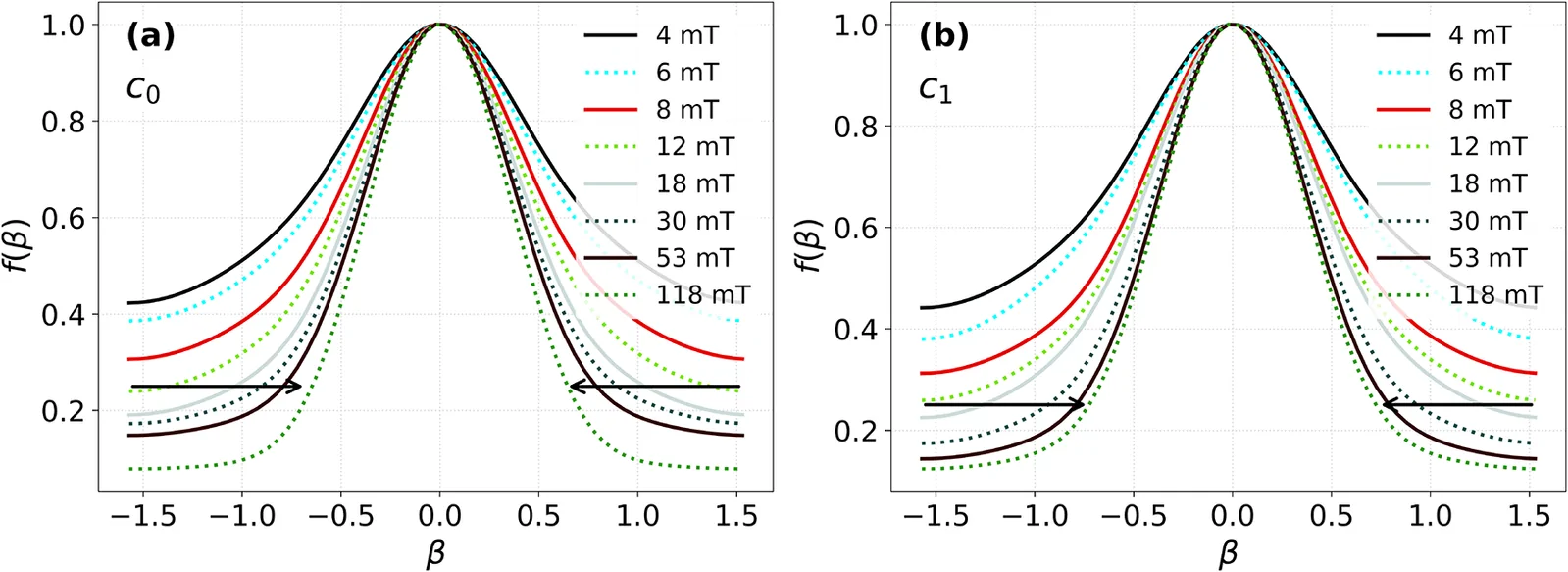
Circular-function approach to the orientation distribution functions *f*(β). Normalized orientation distribution functions are shown for particle concentrations *c*_0_ in panel (a) and *c*_1_ in panel (b). The curves correspond to magnetic-field strengths ranging from 4 mT to 118 mT. All distributions are normalized to a maximum value of 1. The arrows indicate the narrowing of the distributions as the magnetic field strength increases.

While *f*(β) contains all the information on the orientation order, quantifications are often provided through orientation order parameters defined through

16 $$\left\langle P_{n}\right\rangle =\frac{\int_{0}^{\pi /2}P_{n}\left(\cos\beta \right)f\left(\beta \right)\sin\beta d\beta }{\int_{0}^{\pi /2}f\left(\beta \right)\sin\beta d\beta }$$

*P*_n_(cos β) in eq 16 is the *n*th-order Legendre polynomial. For symmetry reasons, *n* is zero or even, i.e., all odd-order terms vanish; *f*(β) need not be normalized (see ref 31 for discussion). The ⟨*P*_*n*_⟩ is given by averaging the orientation of all structural units in the sample. ⟨*P*_2_⟩ and the higher-order coefficients are zero for an isotropic sample. The ⟨*P*_2_⟩ = 1 for structural units perfectly oriented parallel to the reference direction. Comparison of *P*_2_(cos β) and eq 5 shows that the order parameter *S* is equivalent to the coefficient of the second Legendre polynomial ⟨*P*_2_⟩.^36^ For our data, it can be calculated as

17 $$\left\langle P_{2}\right\rangle =S_{\mathrm{circular}}=\frac{\int_{0}^{\pi /2}\frac{1}{2}\left(3\cos^{2}\beta -1\right)\left(a_{0}+a_{2}\cos^{2}\beta +a_{8}\cos^{8}\beta \right)\sin\beta d\beta }{\int_{0}^{\pi /2}\left(a_{0}+a_{2}\cos^{2}\beta +a_{8}\cos^{8}\beta \right)\sin\beta d\beta }$$

After integration, this simplifies to

18 $$S_{\mathrm{circular}}=\frac{2\left(33a_{2}+20a_{8}\right)}{55\left(9a_{0}+3a_{2}+a_{8}\right)}$$

with an estimated uncertainty of

19 $$u_{S_{\mathrm{circular}}}=\sqrt{\frac{324\left[{\left(33a_{2}+20a_{8}\right)}^{2}u_{a_{0}}^{2}+9{\left(a_{8}-11a_{0}\right)}^{2}u_{a_{2}}^{2}+{\left(20a_{0}+3a_{2}\right)}^{2}u_{a_{8}}^{2}\right]}{3025{\left(9a_{0}+3a_{2}+a_{8}\right)}^{4}}}$$

Data and curve fit using eq 9 are shown in Figure 8. Therein, the *a*_0_ intercept values are 0.000 ± 0.001 for *c*_0_ and 0.019 ± 0.009 for *c*_1_. The *a*_1_ slope values are 0.077 ± 0.007 (*c*_0_) and 0.065 ± 0.008 (*c*_1_).

**Figure 8:**
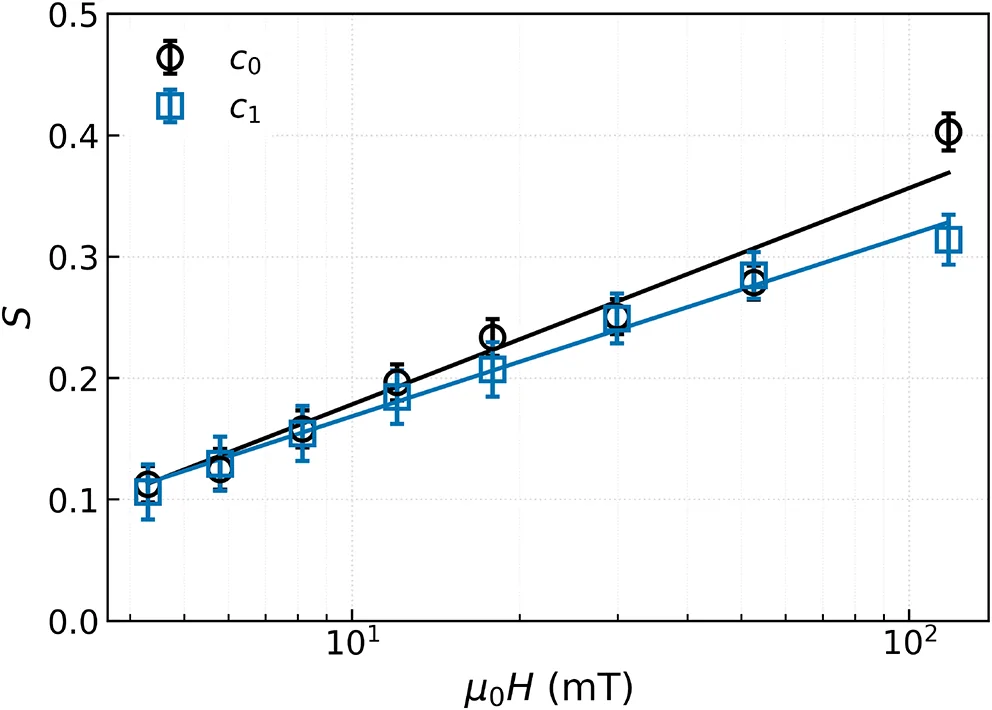
Circular-function approach. Orientation parameter *S* as a function of magnetic-field strength μ_0_ *H* for the particle concentrations *c*_0_ and *c*_1_. Black circles and blue squares denote the data for *c*_0_ and *c*_1_, respectively. Solid lines represent linear fits to the data, yielding slopes of 0.077 ± 0.007 for *c*_0_ and 0.065 ± 0.008 for *c*_1_.

### Model-Free Determination of Nanostructure Orientation

The second-order orientation parameter can also be estimated directly from the azimuthal scattering intensity without assuming a specific orientational distribution function. In this model-free approach, the orientation parameter is calculated as

20 $$S=\frac{1}{2}\left(3\frac{\int_{0}^{\pi /2}I\left(\phi \right)\sin\phi \cos^{2}\phi d\phi }{\int_{0}^{\pi /2}I\left(\phi \right)\sin\phi d\phi }-1\right)$$

This approach was previously applied by Wang et al.^37^ to quantify the alignment of carbon nanotubes in films and by Finnigan et al.^38^ to characterize the orientation of organically modified layered silicates. The ratio of the two integrals in eq 20 corresponds to the mean-square cosine of the azimuthal angle, ⟨cos^2^ ϕ⟩, and was evaluated by numerical integration of the measured azimuthal intensity profiles. Related quantifications of scattering anisotropy and orientational order have also been reported for other anisotropic systems, including ferronematic dispersions,^39^ shear-aligned protein fibers,^40^ and worm-like micelles under shear.^41^

The resulting values of *S* as a function of magnetic field strength for the particle concentrations *c*_0_ and *c*_1_ are shown in Figure S16. Compared with the values obtained from the Maier–Saupe analysis, the model-free values exhibit a larger scatter. Nevertheless, the overall increase of *S* with increasing magnetic field strength is clearly resolved for both concentrations.

To obtain concentration-dependent mean trends, the values determined at different scattering-vector magnitudes were averaged. The averaged values are summarized in Table S3 and were fitted using eq 9, as shown in Figure 9. The fitted intercepts are *a*_0_ = 0.195 ± 0.020 for *c*_0_ and *a*_0_ = 0.139 ± 0.016 for *c*_1_. The corresponding slopes are *a*_1_ = 0.062 ± 0.005 for *c*_0_ and *a*_1_ = 0.050 ± 0.005 for *c*_1_.

**Figure 9:**
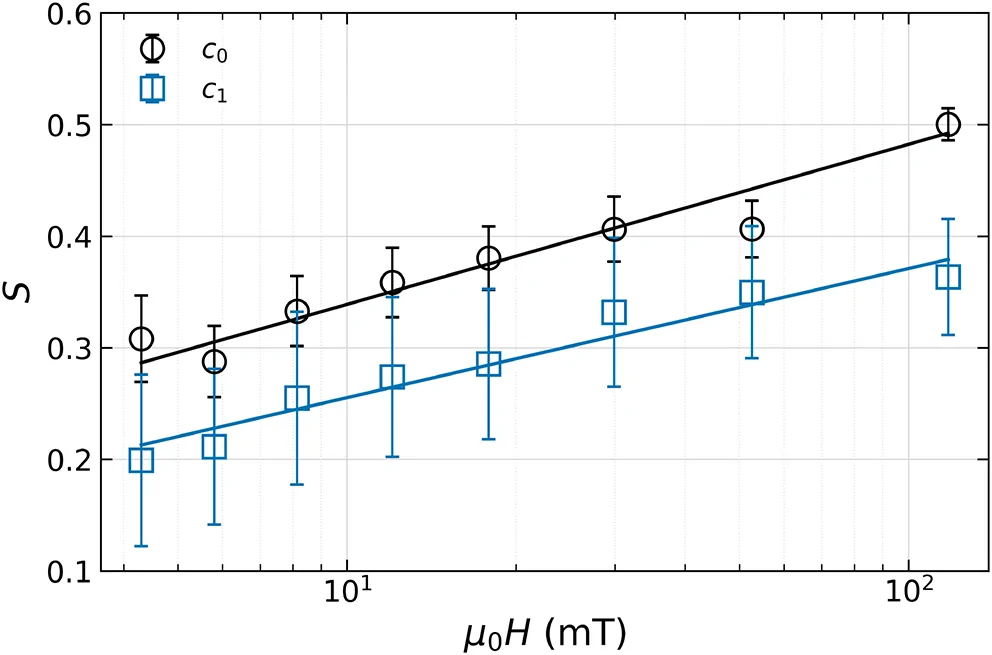
Orientation parameter *S* as a function of the applied magnetic field strength μ_0_ *H*, obtained using the basic model-free analysis. Data are shown for particle concentrations *c*_0_, black circles, and *c*_1_, blue squares. Error bars indicate the uncertainty of the averaged orientation parameter. Solid lines represent linear fits of *S* as a function of ln­(μ_0_ *H*) according to eq 9. The fitted slopes are 0.062 ± 0.005 for *c*_0_ and 0.050 ± 0.005 for *c*_1_, indicating a stronger field-induced increase in orientational order for the higher particle concentration.

Overall, the model-free analysis provides orientation parameters that are qualitatively consistent with the Maier–Saupe-based evaluation, but the absolute values differ noticeably. In particular, the model-free approach does not yield information on the orientational distribution function *f*(β) and is more sensitive to the treatment of the scattering background. In the present analysis, a constant background intensity was subtracted individually from each azimuthal intensity profile. These limitations motivate a comparison with additional analytical methods to further assess the robustness of the extracted orientation parameters.

## Discussion

The three approaches yield a consistent qualitative picture of magnetic-field-induced orientation. However, they differ in the level of model dependence and in the absolute values of the order parameter. The Maier–Saupe analysis provides the most compact description, because the full orientational distribution is represented by a single parameter, *m*, from which the second-order order parameter *S* is obtained analytically. This approach yields smooth, physically plausible orientation distributions that narrow systematically as the magnetic field strength increases. The corresponding *S* values increase approximately linearly with ln­(μ_0_ *H*) for both concentrations, with a larger slope for *c*_0_ than for *c*_1_. This indicates that the higher-concentration sample develops orientational order more efficiently under the applied magnetic field.

The circular-function expansion provides an independent and more flexible description of the orientational distribution. Instead of assuming a Maier–Saupe form, the orientation distribution is expressed as a finite series in even powers of cos β. After constraining the expansion to the coefficients *a*_0_, *a*_2_, and *a*_8_, stable fits were obtained. The resulting distributions show the same field-induced narrowing as observed in the Maier–Saupe analysis. The field dependence of the expansion coefficients provides additional insight: *a*_0_ decreases slightly with field strength, *a*_2_ reaches a maximum at intermediate fields, and *a*_8_ increases strongly at high fields. The much stronger increase of *a*_8_ for *c*_0_ than for *c*_1_ suggests that nonlinear contributions to the orientational distribution are more pronounced at the higher particle concentration. The order parameters obtained from this approach again increase with ln­(μ_0_ *H*) and show a stronger field response for *c*_0_ than for *c*_1_, in agreement with the Maier–Saupe analysis.

The model-free analysis is the least assumption-dependent method, because it directly evaluates the second moment of the measured azimuthal intensity distribution without explicitly reconstructing *f*(β). It therefore provides a useful reference for testing the robustness of the model-based approaches. However, the resulting *S* values show larger scatter and are more sensitive to background subtraction and noise in the azimuthal profiles. In contrast to the Maier–Saupe and circular-function approaches, the model-free method does not provide the full orientational distribution function. Nevertheless, it confirms the central trend that the orientational order increases with magnetic field strength. The concentration dependence is less clearly resolved in the model-free analysis, in which the slopes obtained for *c*_0_ and *c*_1_ (0.062 ± 0.005 and 0.050 ± 0.005, respectively) differ only slightly.

Overall, the comparison shows that the Maier–Saupe and circular-function approaches give mutually consistent descriptions of the field-induced orientation and both resolve a stronger orientational response at the higher concentration. The Maier–Saupe approach is advantageous because of its simple one-parameter description and direct connection to the order parameter. The circular-function expansion is more flexible and reveals higher-order contributions to the orientation distribution, especially through the coefficient *a*_8_. The model-free analysis is useful as an independent check but is less precise for the present data set. For two of the three fitting approaches, the linear regression yielded small non-zero intercepts *a*_0_, indicating a minor deviation from perfectly isotropic order in the absence of an applied field; however, given the small magnitude of *a*_0_ relative to the corresponding slopes, this effect was considered negligible and the simple linear regression was retained for all three approaches. Together, the three methods support the conclusion that ferucarbotran nanoparticles develop increasing orientational order in a static magnetic field and that this process is more pronounced in the native, higher-concentration formulation.

Beyond the specific case of ferucarbotran, the present method comparison underlines the importance of standardized workflows for determining orientational order in nanostructures from anisotropic SAXS data. Consistent treatment of angular definitions, normalization, background subtraction, fitting procedures, and uncertainty propagation is required to make orientation parameters comparable across experiments and laboratories. The agreement between the Maier–Saupe, circular-function, and model-free evaluations suggests that their combined use can provide a robust basis for such standardization.

## Conclusion

In conclusion, the orientation of ferucarbotran multicore iron oxide nanoparticles under a magnetic field was evaluated using SAXS. The initially isotropic scattering patterns became anisotropic as the magnetic field strength increased, indicating a preferential alignment of the nanoparticles. A one-dimensional multicore model accurately captured the main features of the scattering curve and supported orientation analysis. The assessment of orientational order involved three methods: the Maier–Saupe distribution, circular-function expansion, and model-free analysis. Both the Maier–Saupe and circular-function methods showed that the second-order orientational order parameter increases approximately linearly with the natural logarithm of the magnetic field strength, ln­(μ_0_ *H*). This increase was more apparent in the native formulation than in the diluted sample, indicating a concentration-dependent effect. The circular-function analysis also identified nonlinear contributions at the higher concentration. Despite some variability in the model-free approach, it aligned with the overall trend toward increased orientational order at higher magnetic field strength. These findings confirm SAXS as an effective method for quantifying orientational order in magnetic nanoparticle dispersions and suggest the potential for standardized analytical protocols in this area.

## Supplementary Material



## Notes

Experimental setup for SAXS measurements of ferucarbotran in an external magnetic field (Figure S1); magnetic-field calibration as a function of magnet separation distance (Figure S2 and Table S1); mass magnetization curve and selected SAXS field strengths (Figure S3); SAXS intensity contour maps recorded at different magnetic field strengths (Figure S4); Kratky representation of the isotropic SAXS data (Figure S5); Maier–Saupe analysis of azimuthal intensity profiles and corresponding orientational order parameters (Figures S6–S10 and Table S2); circular-function analysis of azimuthal intensity profiles and fit parameters (Figures S11–S15); model-free determination of the orientation parameter (Figure S16 and Table S3) (PDF)
