# Supplementary material for: Comparing Small-Angle X‑ray Scattering Approaches for Quantifying Magnetic-Field-Induced Orientation of Superparamagnetic Iron Oxide Nanoparticles
Source: Langmuir. 2026 Oct 1;42(40):30136–45. doi: 10.1021/acs.langmuir.6c04285 (PMC13674219; doi:10.1021/acs.langmuir.6c04285)
Supplement: Supplementary file 1 [file la6c04285_si_001.pdf]

Supporting Information
Comparing Small-Angle X-ray Scattering Approaches for Quantifying Magnetic-Field-Induced Orientation of Superparamagnetic Iron Oxide Nanoparticles

Andreas F. Thünemann*

Bundesanstalt für Materialforschung und -prüfung (BAM), Unter den Eichen 87, 12205 Berlin, Germany

E-mail: andreas.thuenemann@bam.de

Contents

Experimental **S2**

Maier-Saupe approach **S5**

Circular-functions approach **S10**

Model-free determination of nanostructure orientation **S15**

References **S16**

Experimental

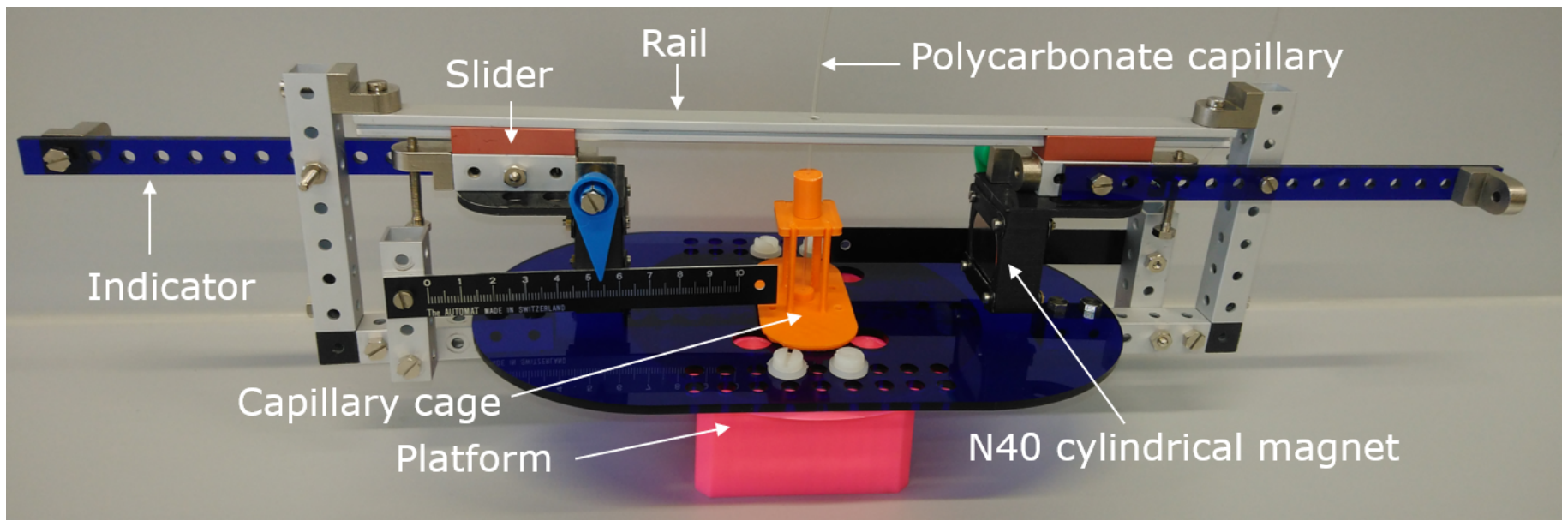

Figure S1: Experimental setup for SAXS measurements of Ferucarbotran in an external magnetic field. A polycarbonate capillary containing the sample is positioned vertically in a sample holder made of poly(lactic acid) (capillary cage in orange). The X-ray beam radiates vertically from the front image plane through the sample. Two permanent magnets (N40 cylindrical magnets) are positioned at a distance, d, on the left- and right-hand side of the capillary using two sliders on a rail. The magnetic field strength can be varied by changing d.

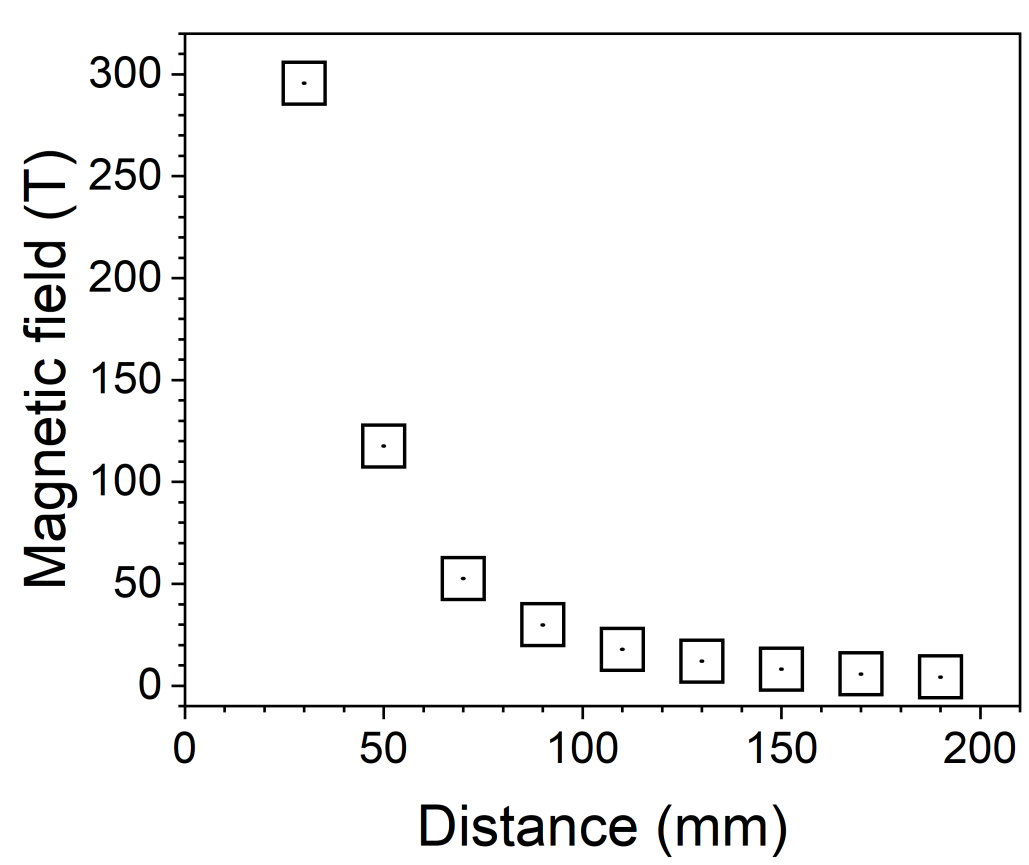

Figure S2: The magnetic field as a function of the distance between the magnets and the capillary center (black squares). The experimental setup is shown in Figure S1.

Table S1: Magnetic field strengths used for the SAXS measurements of ferucarbotran as a function of magnet separation distance. For simplicity, rounded values are reported in the text, while all calculations were performed with the corresponding non-rounded values.

Separation distance (mm)	$\mu_0 H$ (mT)	Rounded $\mu_0 H$ (mT)
190	4.3	4
170	5.8	6
150	8.15	8
130	12.04	12
110	17.87	18
90	29.88	30
70	52.61	53
50	117.62	118
30	295.64	296

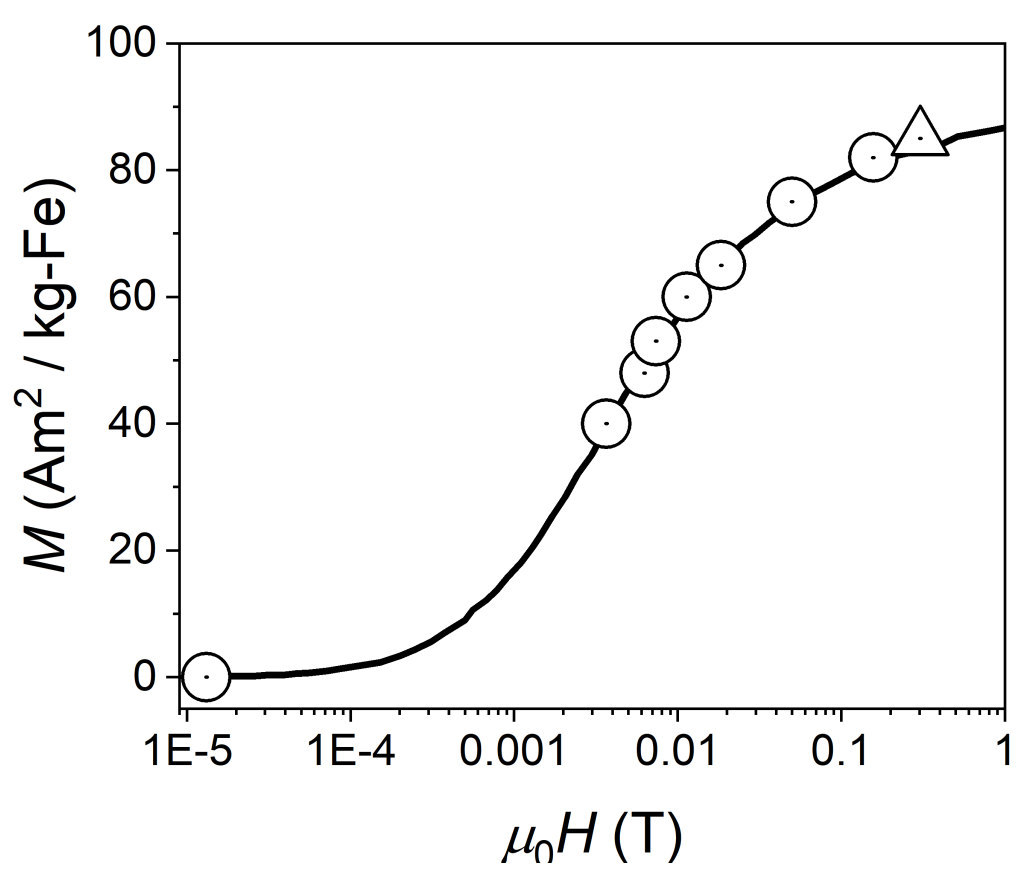

Figure S3: Mass magnetization, M, of ferucarbotran (Resovist) as a function of magnetic field strength, $\mu_0 H$, together with the field values selected for the SAXS measurements (solid line and symbols, respectively). The selected field strengths were 0, 4.3, 5.8, 8.15, 12.04, 17.87, 29.88, 52.61, 117.62, and 295.65 mT. The SAXS data recorded at the highest field strength were omitted, as indicated by the triangle. At this field strength, X-ray transmission decreased during the measurement because magnetic separation caused the particles to move out of the capillary's irradiated volume. The magnetization data for Ferucarbotran were taken from Yoshida et al.[1]

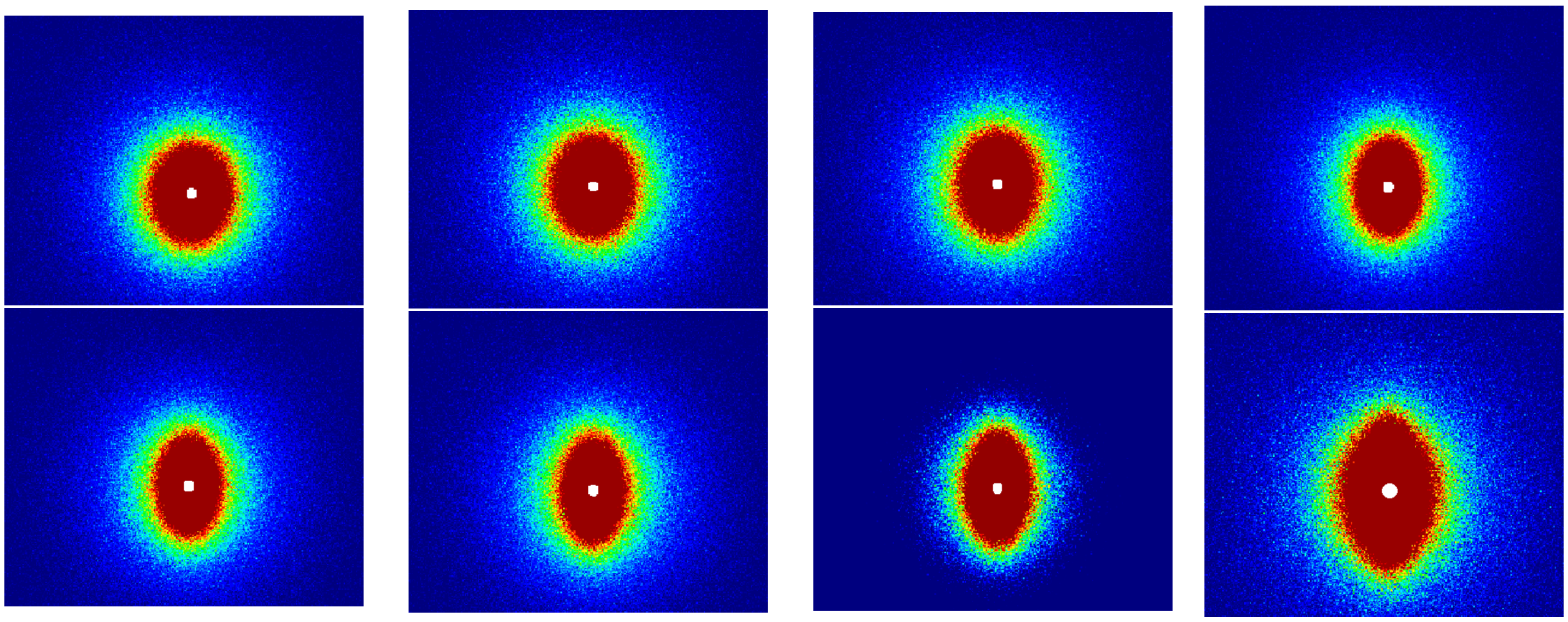

Figure S4: SAXS intensity contour maps of ferucarbotran particles recorded at magnetic field strengths of 4.3, 5.8, 8.15, and 12.04 mT (upper row, from left to right) and 17.87, 29.88, 52.61, and 117.62 mT (lower row, from left to right). The magnetic field direction is horizontal. The particle concentration was c_0.

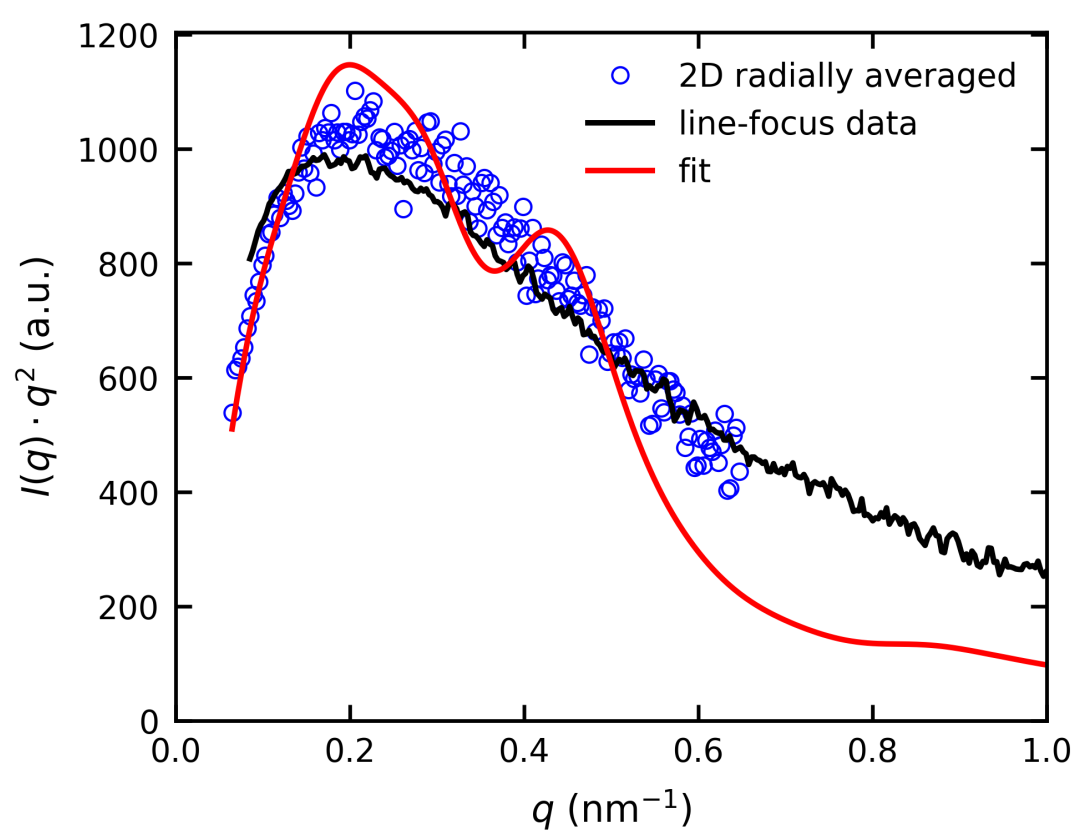

Figure S5: Kratky representation, $I(q) \cdot q^2$, of the isotropic SAXS data of Figure 1 (c_0, zero field). Blue open circles: present 2D data; black line: line-focus data from an earlier study,[2] red line: model fit. The pronounced maximum followed by a shoulder rather than a plateau at high q is consistent with the non-globular, chain-like multicore particle model used in the fit.

Maier-Saupe approach

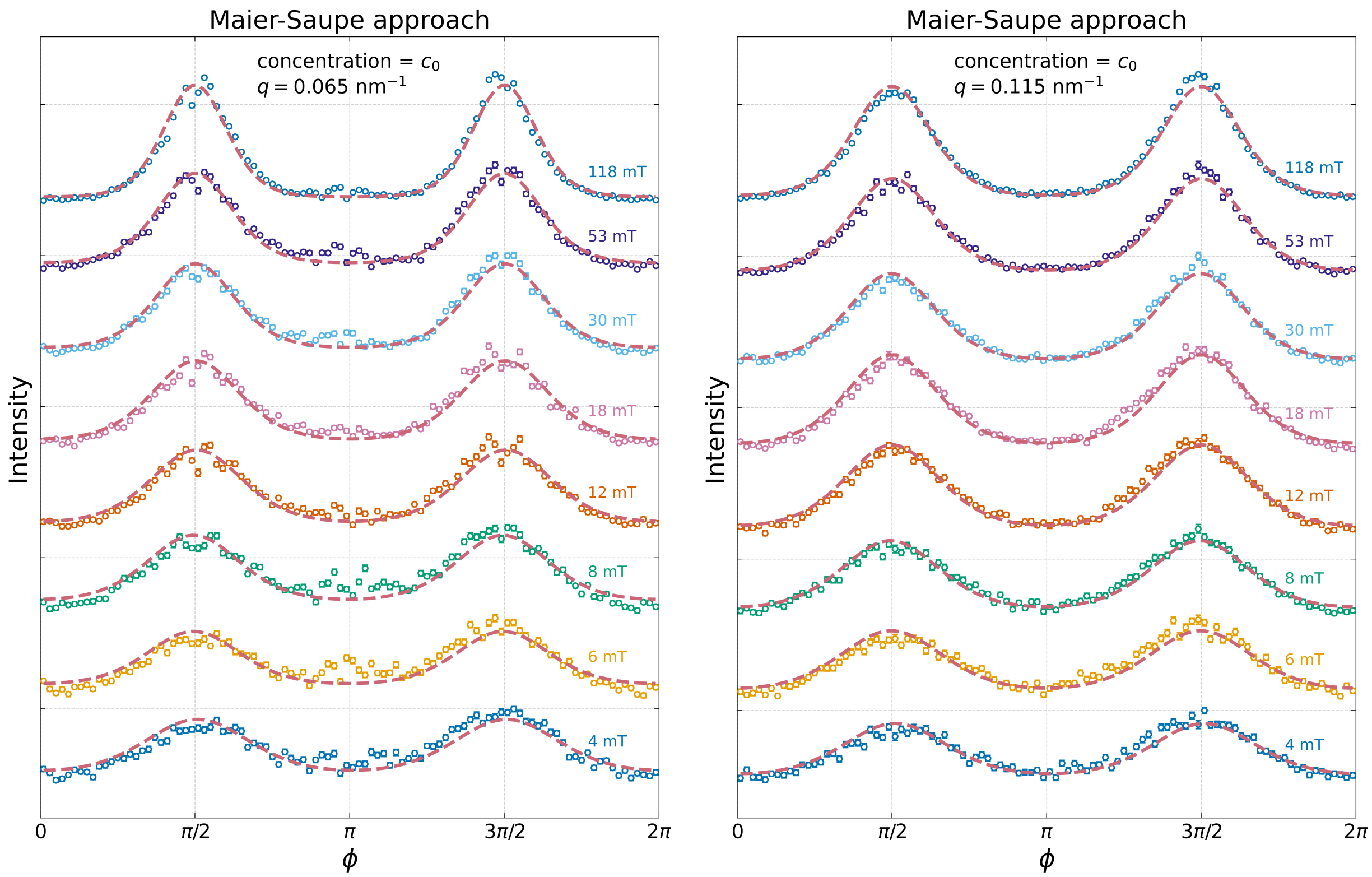

Figure S6: Azimuthal intensity profiles analyzed using the Maier–Saupe approach for samples at concentration c_0. Scattering intensity is plotted as a function of azimuthal angle ϕ at fixed scattering vectors of $q = 0.065$ nm^{-1} and $q = 0.115$ nm^{-1} for applied magnetic fields between 4 and 118 mT, as indicated. Symbols represent the experimental intensity profiles, and red solid lines denote Maier–Saupe fits. The profiles are vertically offset for clarity. Dashed vertical lines mark the principal azimuthal peak positions at $\phi = \pi/2$ and $3\pi/2$, highlighting the field-induced angular anisotropy of the scattering pattern.

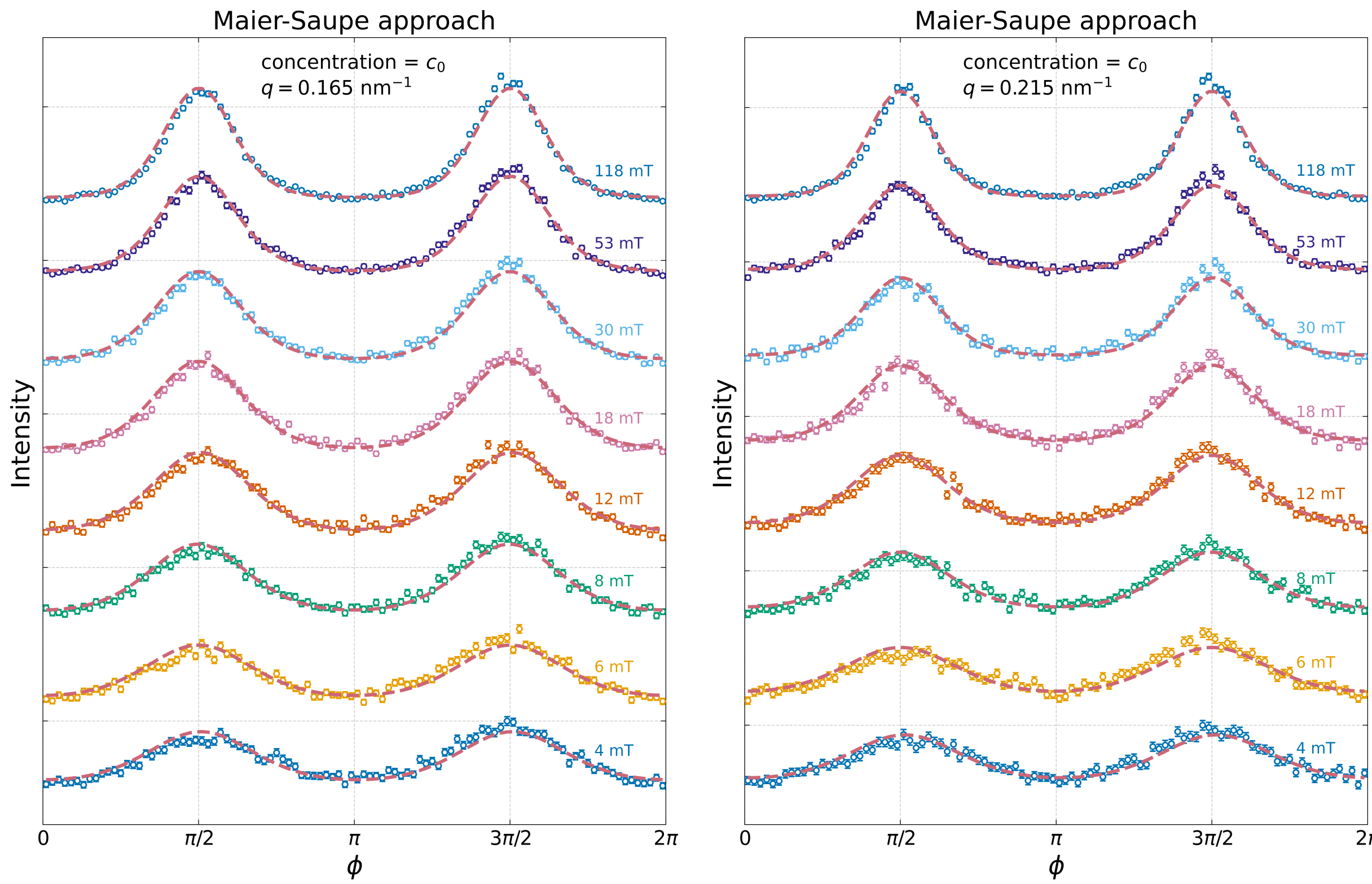

Figure S7: Azimuthal intensity profiles analyzed using the Maier–Saupe approach for samples at concentration c_0. Scattering intensity is plotted as a function of azimuthal angle ϕ at fixed scattering vectors of $q = 0.165$ nm^{-1} and $q = 0.215$ nm^{-1} for applied magnetic fields between 4 and 118 mT, as indicated. Symbols represent the experimental intensity profiles, and red solid lines denote Maier–Saupe fits. The profiles are vertically offset for clarity. Dashed vertical lines mark the principal azimuthal peak positions at $\phi = \pi/2$ and $3\pi/2$, highlighting the field-induced angular anisotropy of the scattering pattern.

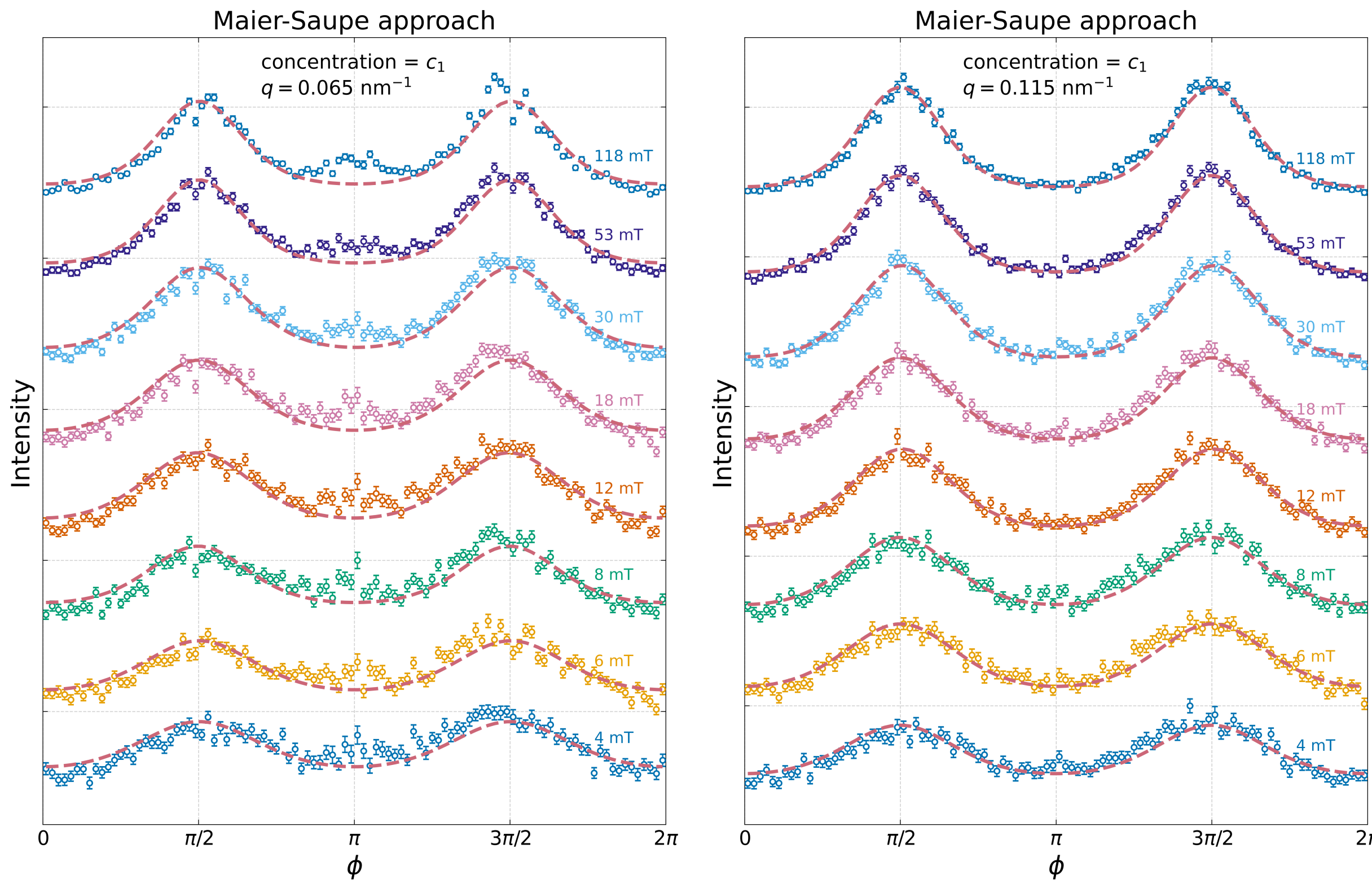

Figure S8: Azimuthal intensity profiles analyzed using the Maier–Saupe approach for samples at concentration c_1. Scattering intensity is plotted as a function of azimuthal angle ϕ at fixed scattering vectors of $q = 0.065$ nm^{-1} and $q = 0.115$ nm^{-1} for applied magnetic fields between 4 and 118 mT, as indicated. Symbols represent the experimental intensity profiles, and red solid lines denote Maier–Saupe fits. The profiles are vertically offset for clarity. Dashed vertical lines mark the principal azimuthal peak positions at $\phi = \pi/2$ and $3\pi/2$, highlighting the field-induced angular anisotropy of the scattering pattern.

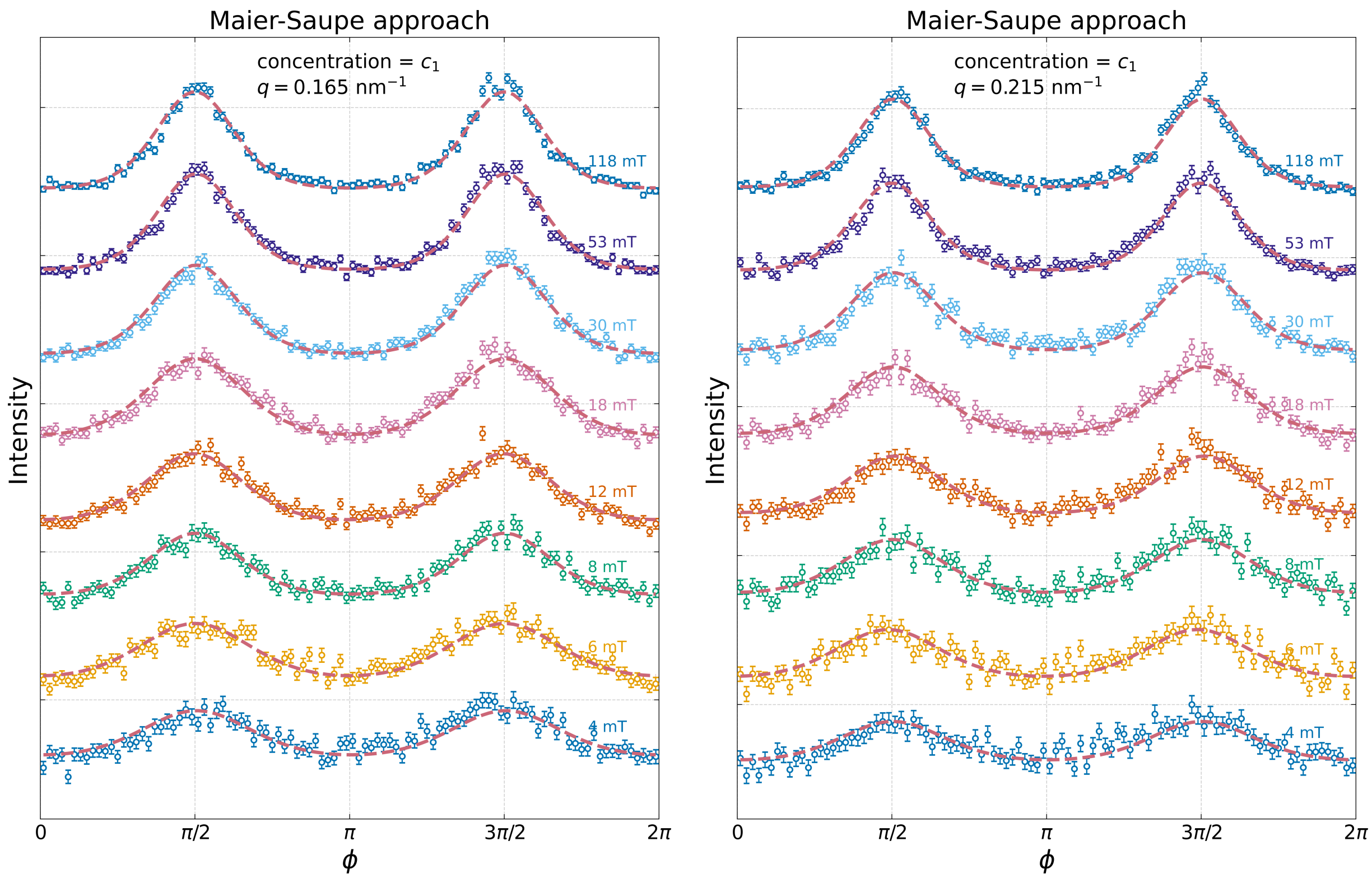

Figure S9: Azimuthal intensity profiles analyzed using the Maier–Saupe approach for samples at concentration c_1. Scattering intensity is plotted as a function of azimuthal angle ϕ at fixed scattering vectors of $q = 0.165$ nm^{-1} and $q = 0.215$ nm^{-1} for applied magnetic fields between 4 and 118 mT, as indicated. Symbols represent the experimental intensity profiles, and red solid lines denote Maier–Saupe fits. The profiles are vertically offset for clarity. Dashed vertical lines mark the principal azimuthal peak positions at $\phi = \pi/2$ and $3\pi/2$, highlighting the field-induced angular anisotropy of the scattering pattern.

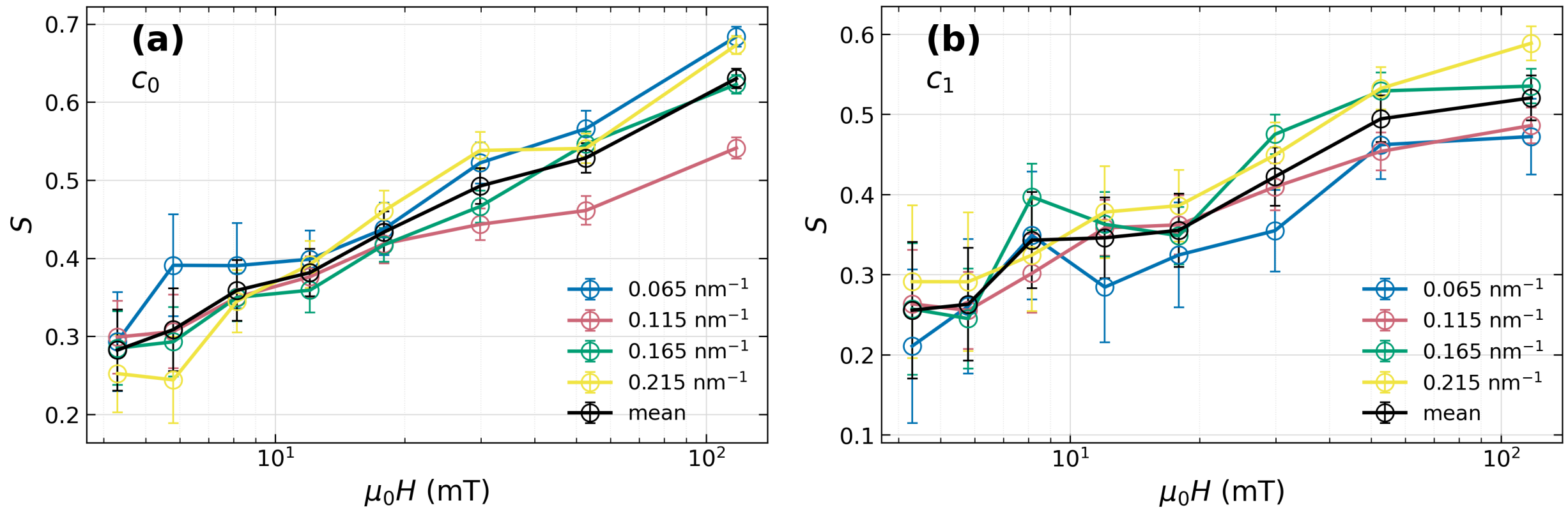

Figure S10: Magnetic-field dependence of the orientational order parameter S obtained using the Maier–Saupe approach for particle concentrations c_0 and c_1. Panels (a) and (b) show the results for c_0 and c_1, respectively. Symbols represent the experimentally determined values at four scattering vectors, $q = 0.065$ nm^{-1}, 0.115 nm^{-1}, 0.165 nm^{-1}, and 0.215 nm^{-1}, shown in blue, red, green, and yellow, respectively. The black symbols indicate the average values calculated from these four q-dependent data sets. The averaged values were used for the subsequent evaluation.

Table S2: Summary of the magnetic field strength M, Maier–Saupe parameter m, and orientational order parameter S for particle concentrations c_0 and c_1. The left-hand and right-hand tables show the results for c_0 and c_1, respectively.

M (mT)	m	S
4.3	1.9 ± 0.34	0.28± 0.05
5.8	2.08± 0.36	0.31± 0.05
8.15	2.42± 0.27	0.36± 0.04
12.04	2.58± 0.22	0.38± 0.03
17.87	3.05± 0.21	0.45± 0.02
29.88	3.4 ± 0.18	0.49± 0.02
52.61	3.85± 0.19	0.54± 0.01
117.62	4.93± 0.17	0.64± 0.01

M (mT)	m	S
4.3	1.73± 0.56	0.26± 0.09
5.8	1.78± 0.46	0.26± 0.07
8.15	2.31± 0.41	0.34± 0.06
12.04	2.39± 0.35	0.35± 0.05
17.87	2.39± 0.32	0.36± 0.05
29.88	2.88± 0.27	0.42± 0.04
52.61	3.45± 0.24	0.49± 0.03
117.62	3.69± 0.25	0.52± 0.03

Circular-functions approach

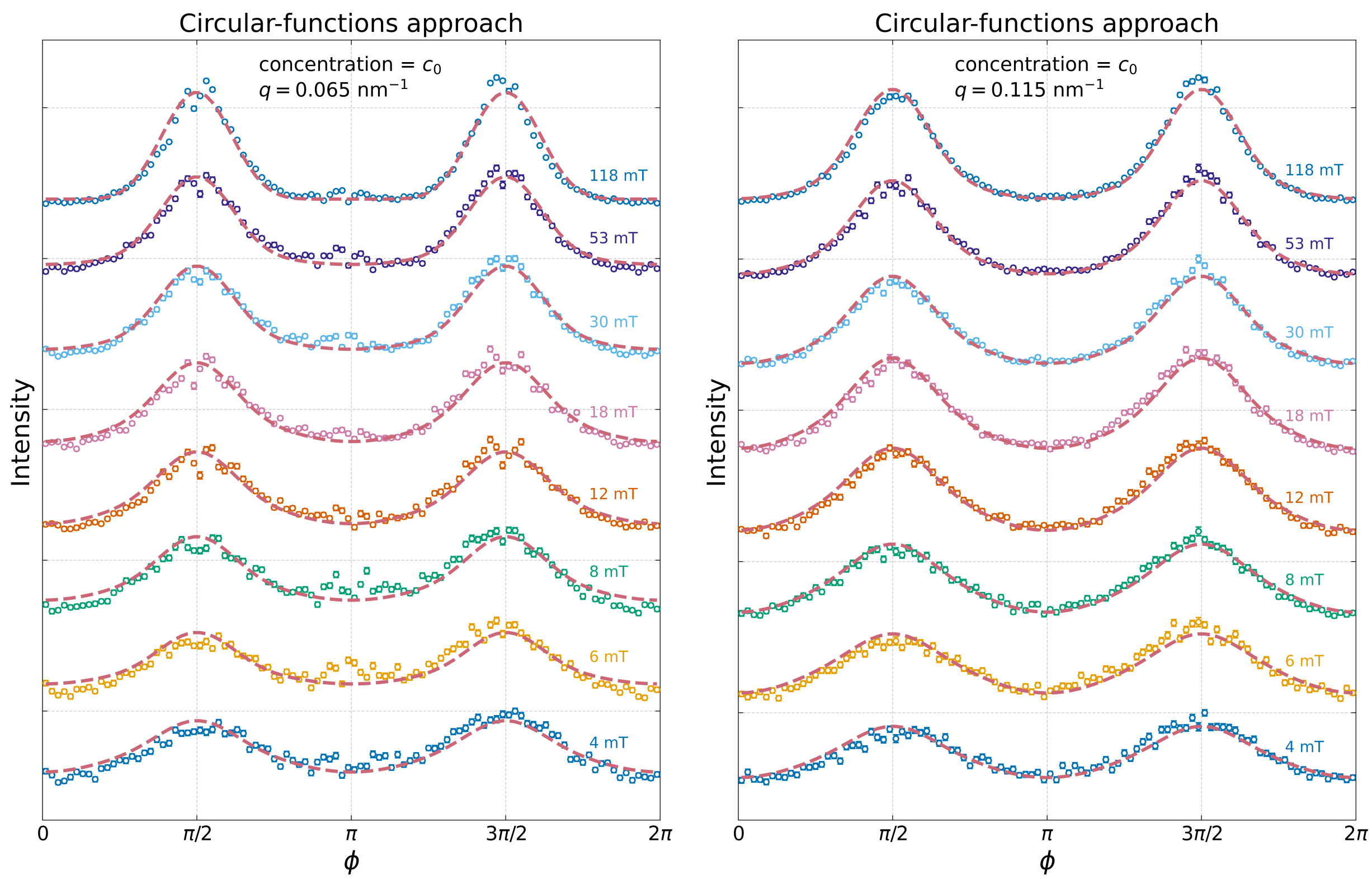

Figure S11: Azimuthal intensity profiles analyzed using the circular function approach for samples at concentration c_0. Scattering intensity is plotted as a function of azimuthal angle ϕ at fixed scattering vectors of $q = 0.065$ nm^{-1} and $q = 0.115$ nm^{-1} for applied magnetic fields between 4 and 118 mT, as indicated. Symbols represent the experimental intensity profiles, and red solid lines denote circular function fits. The profiles are vertically offset for clarity. Dashed vertical lines mark the principal azimuthal peak positions at $\phi = \pi/2$ and $3\pi/2$, highlighting the field-induced angular anisotropy of the scattering pattern.

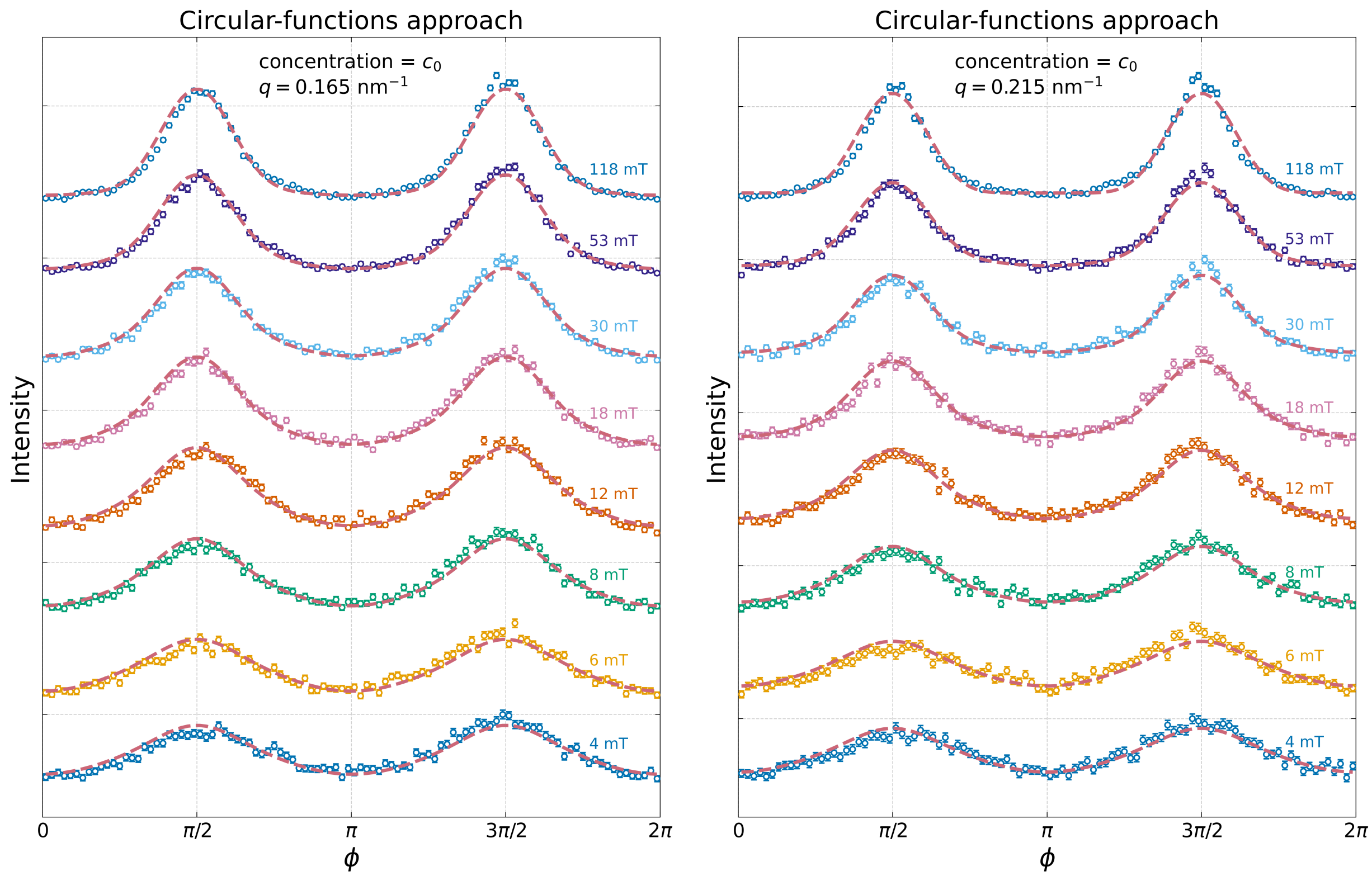

Figure S12: Azimuthal intensity profiles analyzed using the circular function approach for samples at concentration c_0. Scattering intensity is plotted as a function of azimuthal angle ϕ at fixed scattering vectors of $q = 0.165$ nm^{-1} and $q = 0.215$ nm^{-1} for applied magnetic fields between 4 and 118 mT, as indicated. Symbols represent the experimental intensity profiles, and red solid lines denote circular function fits. The profiles are vertically offset for clarity. Dashed vertical lines mark the principal azimuthal peak positions at $\phi = \pi/2$ and $3\pi/2$, highlighting the field-induced angular anisotropy of the scattering pattern.

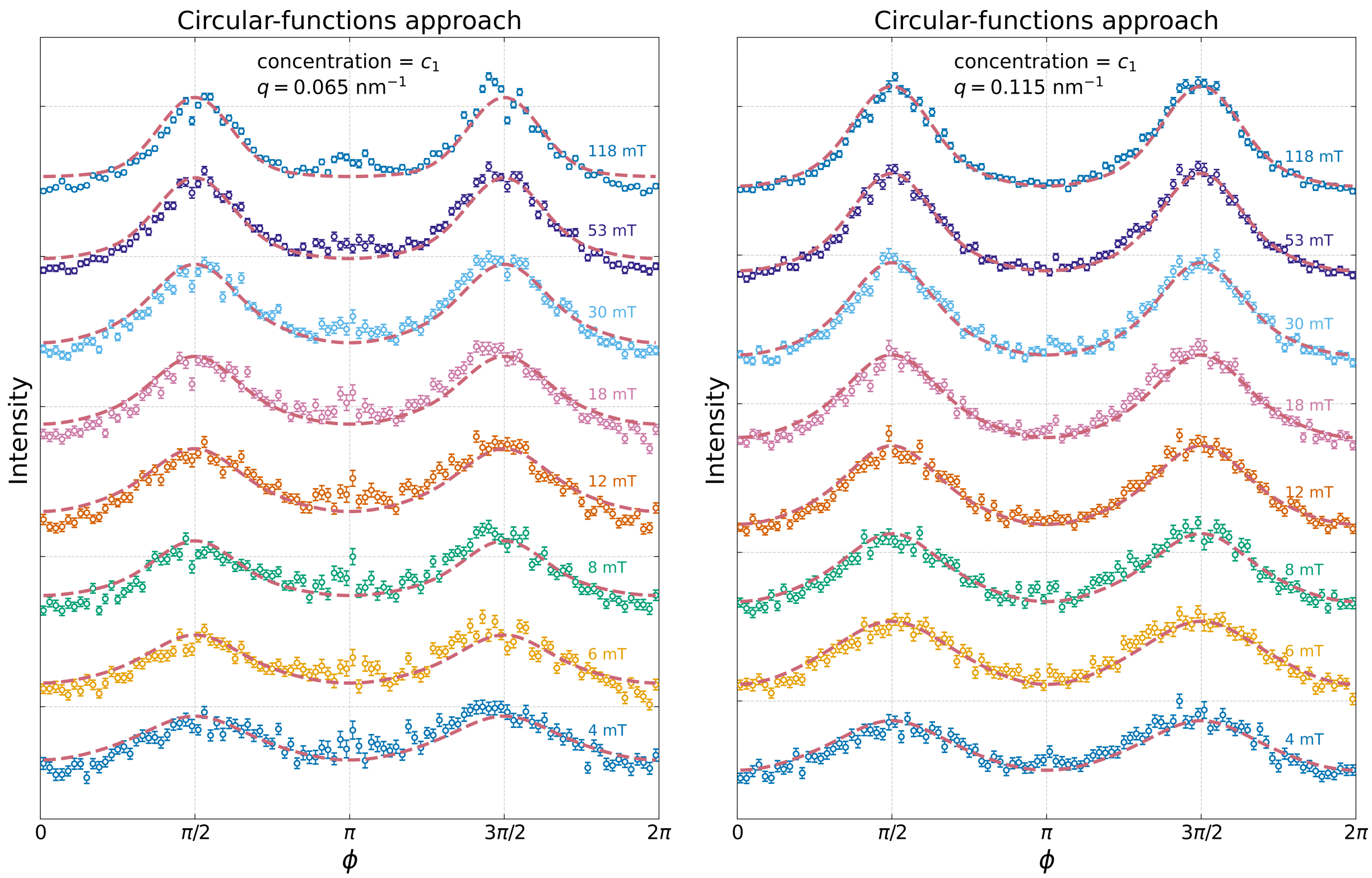

Figure S13: Azimuthal intensity profiles analyzed using the circular function approach for samples at concentration c_1. Scattering intensity is plotted as a function of azimuthal angle ϕ at fixed scattering vectors of $q = 0.065$ nm^{-1} and $q = 0.115$ nm^{-1} for applied magnetic fields between 4 and 118 mT, as indicated. Symbols represent the experimental intensity profiles, and red solid lines denote circular function fits. The profiles are vertically offset for clarity. Dashed vertical lines mark the principal azimuthal peak positions at $\phi = \pi/2$ and $3\pi/2$, highlighting the field-induced angular anisotropy of the scattering pattern.

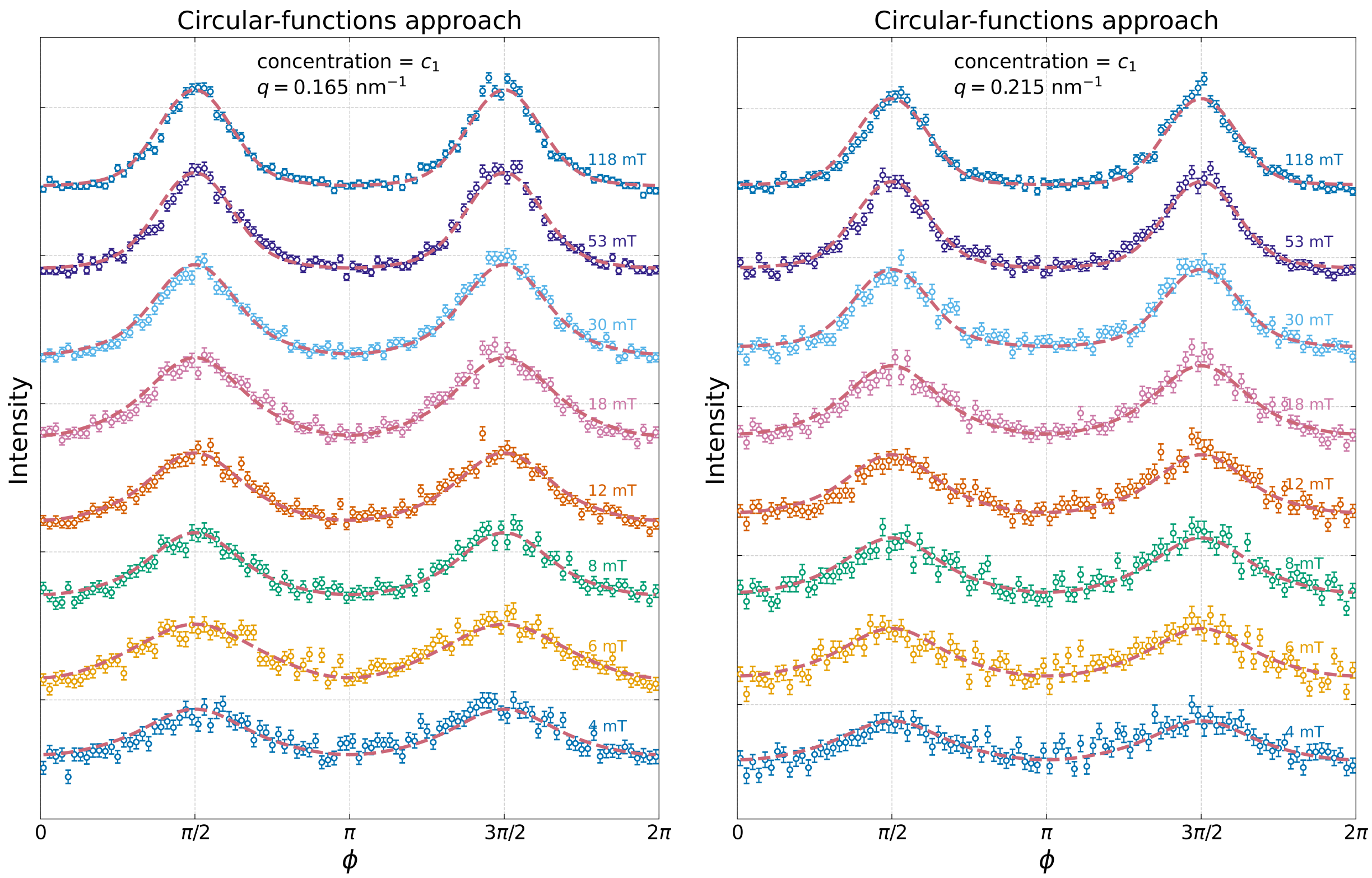

Figure S14: Azimuthal intensity profiles analyzed using the circular function approach for samples at concentration c_1. Scattering intensity is plotted as a function of azimuthal angle ϕ at fixed scattering vectors of $q = 0.165$ nm^{-1} and $q = 0.215$ nm^{-1} for applied magnetic fields between 4 and 118 mT, as indicated. Symbols represent the experimental intensity profiles, and red solid lines denote circular function fits. The profiles are vertically offset for clarity. Dashed vertical lines mark the principal azimuthal peak positions at $\phi = \pi/2$ and $3\pi/2$, highlighting the field-induced angular anisotropy of the scattering pattern.

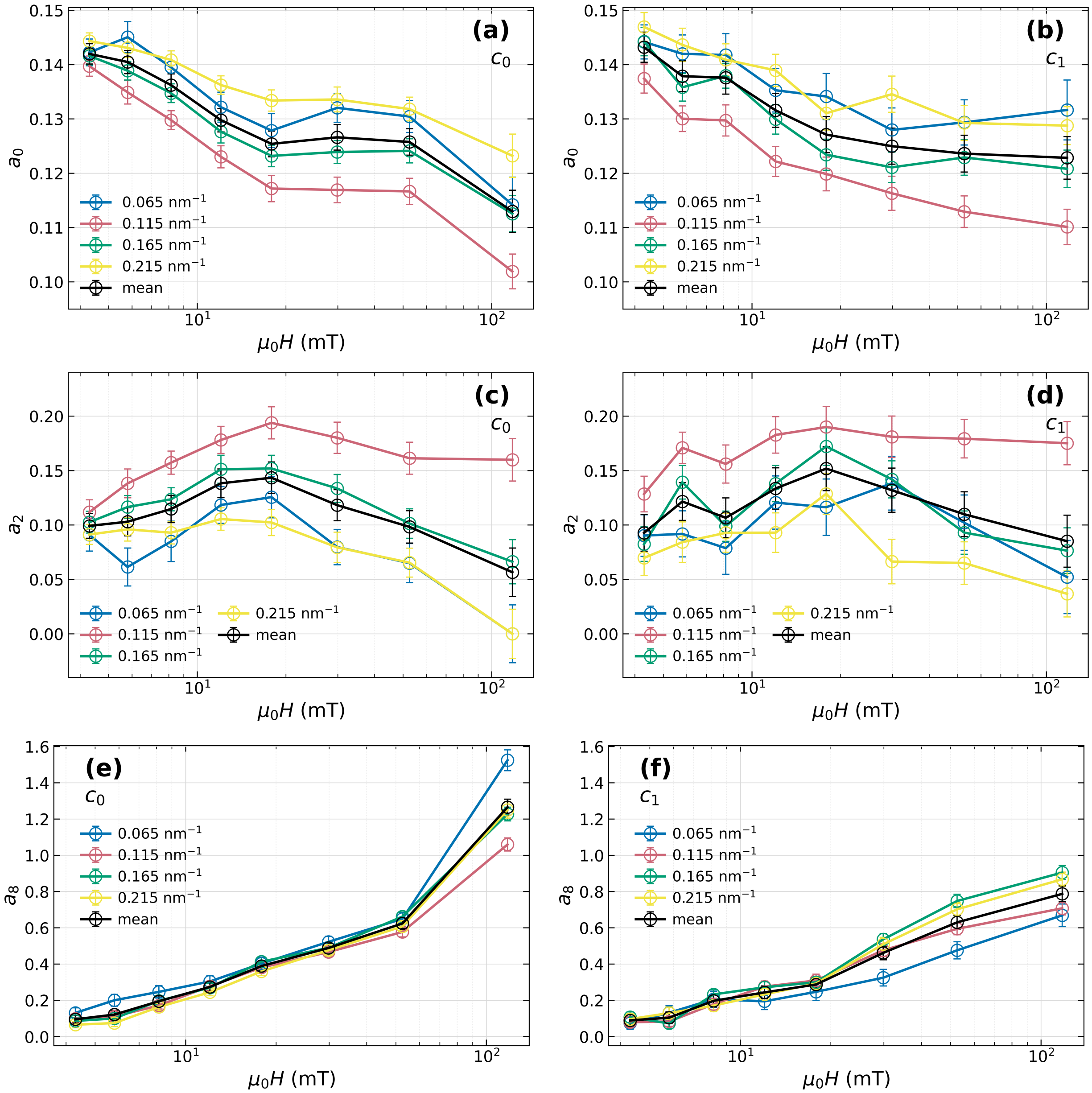

Figure S15: Magnetic-field dependence of the fit parameters a_0, a_2, and a_8 obtained using the circular-functions approach for particle concentrations c_0 and c_1. Panels (a,b), (c,d), and (e,f) show the parameters a_0, a_2, and a_8, respectively. The left-hand panels correspond to concentration c_0, whereas the right-hand panels correspond to concentration c_1. Data are shown for four scattering vectors, $q = 0.065\ \text{nm}^{-1}$, $0.115\ \text{nm}^{-1}$, $0.165\ \text{nm}^{-1}$, and $0.215\ \text{nm}^{-1}$, indicated in blue, red, green, and yellow, respectively. Black symbols represent the average over the four q values. The averaged values were used for the subsequent evaluation.

Model-free determination of nanostructure orientation

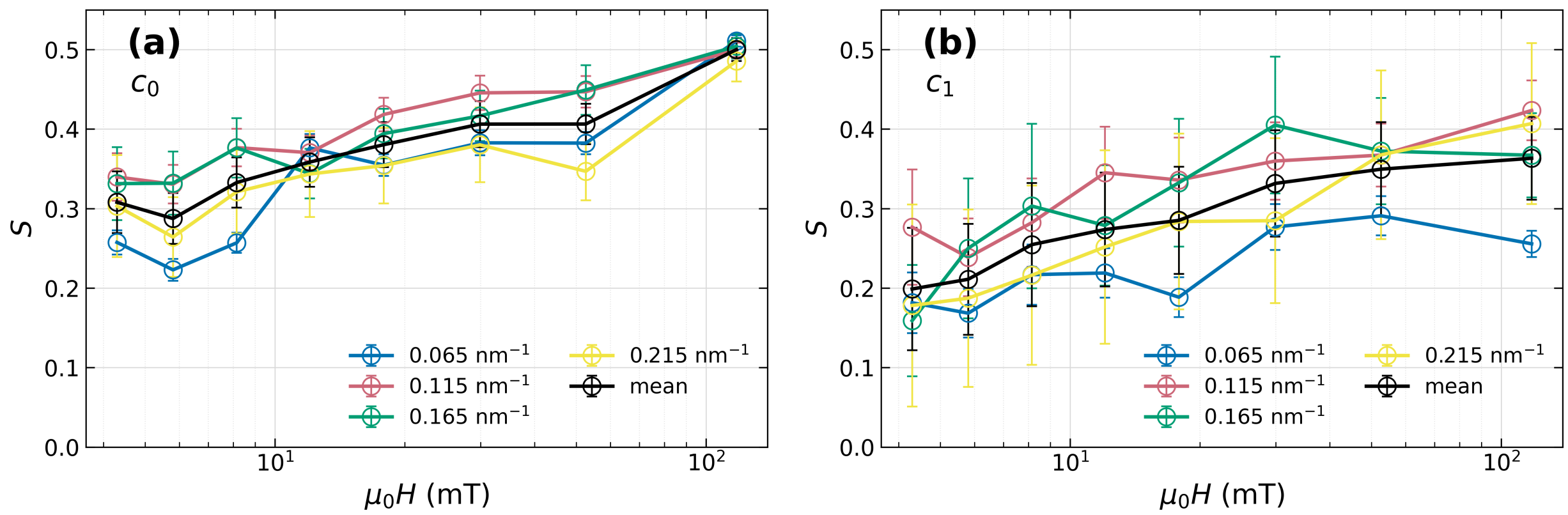

Figure S16: Magnetic-field dependence of the orientation parameter S obtained from a basic model-free analysis for particle concentrations c_0 and c_1. Panels (a) and (b) show the results for c_0 and c_1, respectively. Symbols represent measurements at four scattering-vector magnitudes: $q = 0.065$ nm^{-1} blue, 0.115 nm^{-1} red, 0.165 nm^{-1} green, and 0.215 nm^{-1} yellow. The black symbols indicate the corresponding averaged values across the investigated q range. Error bars denote the uncertainties of the fitted orientation parameter. For both concentrations, S generally increases with increasing magnetic field strength $\mu_0 H$, indicating enhanced field-induced orientational alignment. The averaged values were used for the subsequent quantitative evaluation.

Table S3: Orientation parameter S obtained from the basic model-free analysis as a function of the applied magnetic field strength $\mu_0 H$ for particle concentrations c_0 and c_1. Values are reported as mean ± uncertainty.

$\mu_0 H$ (mT)	$S(c_0)$	$S(c_1)$
4.30	0.31 ± 0.04	0.20 ± 0.08
5.80	0.29 ± 0.03	0.21 ± 0.07
8.15	0.33 ± 0.03	0.25 ± 0.08
12.04	0.36 ± 0.03	0.27 ± 0.07
17.87	0.38 ± 0.03	0.29 ± 0.07
29.88	0.41 ± 0.03	0.33 ± 0.07
52.61	0.41 ± 0.03	0.35 ± 0.06
117.62	0.50 ± 0.01	0.36 ± 0.05

References

(1) Yoshida, T.; Nakamura, T.; Higashi, O.; Enpuku, K. Magnetic fractionation and characterization of magnetic nanoparticles for magnetic particle imaging. *Japanese Journal of Applied Physics* **2018**, *57*, 080302.

(2) Thünemann, A. F.; Rolf, S.; Knappe, P.; Weidner, S. In Situ Analysis of a Bimodal Size Distribution of Superparamagnetic Nanoparticles. *Analytical Chemistry* **2009**, *81*, 296–301.
